# A novel Asfarvirus-like virus identified as a potential cause of mass mortality of abalone

**DOI:** 10.1038/s41598-020-61492-3

**Published:** 2020-03-12

**Authors:** Tomomasa Matsuyama, Tomokazu Takano, Issei Nishiki, Atushi Fujiwara, Ikunari Kiryu, Mari Inada, Takamitsu Sakai, Sachiko Terashima, Yuta Matsuura, Kiyoshi Isowa, Chihaya Nakayasu

**Affiliations:** 1 National Research Institute of Aquaculture, Japan Fisheries Research and Education Agency, Research Center for Fish Diseases, Minami-Ise, Mie 516-0193 Japan; 2 National Research Institute of Fisheries Science, Japan Fisheries Research and Education Agency, Research Center for Bioinformatics and Biosciences, Yokohama, Kanagawa 236-8648 Japan; 3 Japan Fisheries Research and Education Agency, Minato Mirai, Nishi-ku, Yokohama, Kanagawa 220-6115 Japan; 4 National Research Institute of Aquaculture, Japan Fisheries Research and Education Agency, Diagnosis and Training Center for Fish Diseases, Minami-Ise, Mie 516-0193 Japan; 5 Mie Prefectural Sea Farming Center, Owase, Japan

**Keywords:** Virology, Marine biology, Viral infection

## Abstract

A novel Asfarvirus-like virus is proposed as the etiological agent responsible for mass mortality in abalone. The disease, called abalone amyotrophia, originally was recognized in the 1980s, but efforts to identify a causative agent were unsuccessful. We prepared a semi-purified fraction by nuclease treatment and ultracentrifugation of diseased abalone homogenate, and the existence of the etiological agent in the fraction was confirmed by a challenge test. Using next-generation sequencing and PCR-based epidemiological surveys, we obtained a partial sequence with similarity to a member of the family Asfarviridae. BLASTP analysis of the predicted proteins against a virus database resulted in 48 proteins encoded by the novel virus with top hits against proteins encoded by African swine fever virus (ASFV). Phylogenetic analyses of predicted proteins of the novel virus confirmed that ASFV represents the closest relative. Comparative genomic analysis revealed gene-order conservation between the novel virus and ASFV. *In situ* hybridization targeting the gene encoding the major capsid protein of the novel virus detected positive signals only in tissue from diseased abalone. The results of this study suggest that the putative causative agent should be considered a tentative new member of the family Asfarviridae, which we provisionally designate abalone asfa-like virus (AbALV).

## Introduction

African swine fever virus (ASFV) is the causative agent of African swine fever (ASF). The virus causes a hemorrhagic fever with high mortality, with rates approaching 100% in domestic pigs^1^. The virus infects domestic pigs and their relatives and ticks^2^. ASF outbreaks had been recorded in Africa and Europe, but in recent years the disease has spread to China, Vietnam, Cambodia, Mongolia, Hong Kong, and Korea, becoming a threat to the swine industry worldwide^3^. ASFV is a member of nucleocytoplasmic large DNA viruses (NCLDVs) with an average diameter of 200 nm. Although some related viruses, such as faustovirus^4^, kaumoebavirus^5^, and pacmanvirus^6^, have been reported, ASFV is the only member of the Asfarviridae family^7^. In the present paper, we describe a virus likely to be the closest ASFV relative found to date; this novel virus was isolated as the presumptive causative agent of abalone amyotrophia.

Mass mortalities of abalone have been reported since the early 1980s, during seed production in Japan. The disease was designated abalone amyotrophia because diseased abalone develop muscle atrophy in the mantle and foot^8^. Diseased abalone show reduced ability to adhere to the substrate, and some diseased abalone exhibit incisions on the front margin of the shell and brown pigmentation inside of the shell^9^. Histopathological evaluation has revealed the presence of abnormal cell masses that are produced extensively, primarily in the ganglion and peripheral nerve of the foot muscle^9^. Cumulative mortality can reach 50% and higher^10^. Abalone herpesvirus (AbHV)^11,12^ and abalone shriveling syndrome-associated virus (AbSV)^13^ also cause mortality accompanied by amyotrophia, but the latent period differs between these two viral infections and abalone amyotrophia. Abalone infected with AbHV^14^ and AbSV^13^ show signs of disease onset or mortality several days after artificial infection compared with approximately 2 months after challenge in abalone amyotrophia^15^. The causative agent is thought to be a virus, based on the demonstrated infectivity of a 0.22-μm-filtered homogenate derived from affected juvenile abalone^10,16^. Although radiation of the water supply with ultraviolet light successfully prevents the occurrence of this disease^17^, the disease still occurs occasionally in facilities that do not employ UV sterilizers.

Shellfish are an important food source for people in many countries, but resources are sometimes decreased due to infectious diseases^18–26^. Pathogen identification for shellfish diseases, especially for viral diseases, is difficult because of the lack of cell lines available for virus isolation and serological methods are hampered by the absence of antibodies and pathogen-specific immune mechanisms in mollusks^27^. For these reasons, searches for pathogenic viruses have relied primarily on electron microscopic observation^25^. Indeed, tissues from diseased abalone have been screened for pathogenic viruses by electron microscopy, but those studies were not successful.

Subsequently, Nakatsugawa *et al*.^28^ succeeded in isolating and culturing spherical viruses with a diameter of about 120 nm from affected abalone using primary cultured hemolymph cells. The resemble virus also was cultured from affected abalone obtained from different areas, but these virions did not show pathogenicity in infection tests^28^. Virus-like particles with a diameter of 100 nm were observed in cells near the nerve trunk of affected abalone, but the pathogenicity of these particles was not demonstrated^29^. Virus-like particles with a diameter of 50–60 nm were observed in secondary lysosomes in the cytoplasm of cells of affected abalone^9^. These authors described that these particles were not likely to be the etiological agent because the size of the causative agent was estimated to be between 100–220 nm by filtration experiments^9,16^.

Recently, new viruses^30–33^ and unculturable bacteria^34^ that infect shellfish have been found using next-generation sequencing (NGS) technologies. Therefore, we applied NGS in the search for the causative agent of abalone amyotrophia. Samples of diseased abalone were collected from hatcheries where mass mortalities occurred. We analyzed the pathogenic semi-purified fraction prepared from diseased abalone homogenate by NGS. Sequences specific to diseased abalone were selected by PCR-based epidemiological survey. As we report here, the presumptive causative agent of abalone amyotrophia was identified as a virus with genomic similarity to ASFV; we propose provisionally designating the new virus as abalone asfa-like virus (AbALV).

## Results

### Infection test

**Figure 1:**
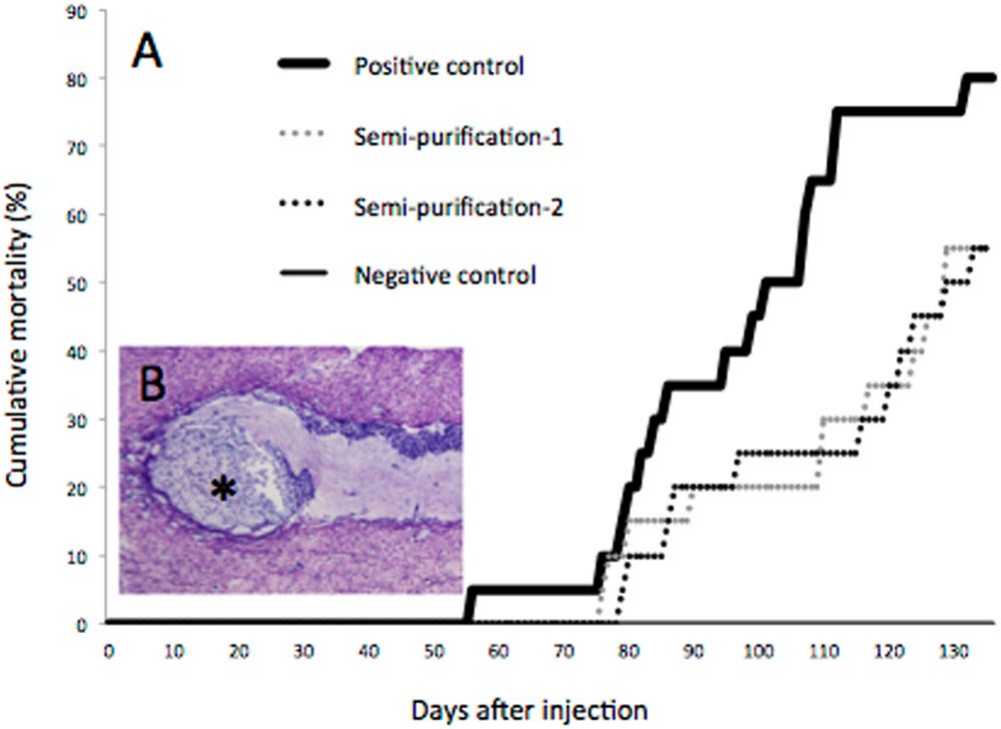
Results of the artificial infection tests. (**A**) Cumulative mortalities of black abalone (*Haliotis discus discus*) in experimental infections. Two groups of abalone were challenged with semi-purified fractions by intramuscular injection (dotted line). The positive control group (bold line) and negative control group (fine line) were treated in the same way with homogenates of diseased and healthy abalone, respectively. (**B**) Histological observation of a survivor from one of the semi-purified fraction-injected groups. Note abnormal cell masses (*). Black bar indicates 50 µm.

An infection test was conducted using healthy black abalone (*Haliotis discus discus*) to confirm the presence of an etiological agent in a semi-purified fraction that had been prepared from a homogenate of diseased black abalone. In the positive control group (which was injected with the homogenate of a diseased abalone), the mortality began on the 56th day post-injection (dpi), with cumulative mortality reaching 80% at 136 dpi (Fig. 1A). In two groups inoculated with the semi-purified fractions from diseased abalone, deaths began at 76 and 79 dpi, with the cumulative mortality rate reaching 55% at 136 dpi in both groups. In contrast, no mortalities occurred in the negative control group, in which abalone were inoculated with the homogenate of healthy abalone. Cumulative mortality was significantly higher in the positive control group and semi-purified fraction-injected groups than in the negative control group (Fisher’s exact test, *p* < 0.01). Among the surviving abalone, abnormal cell masses, which are characteristic of the disease, were observed only in animals of the positive control group and semi-purified fraction-injected groups (Fig. 1B). The semi-purified fraction was used for shotgun sequencing because results of the infection test indicated the presence of the etiological agent in this fraction.

### DNA shotgun sequencing

In the genome sequence of the semi-purified fraction prepared from the diseased abalone, 6.51 × 10^7^ reads consisting of 8.16 × 10^9^ bp were obtained. In the genome sequence of the healthy abalone, 1.86 × 10^8^ reads consisting of 2.55 × 10^10^ bp were obtained. Assembly was performed after pooling the reads from the semi-purified fraction and those from healthy abalone, yielding a total of 5,531 scaffolds and singletons (mean coverage depth 695 ± 392). Of the total scaffolds and singletons, 2,160 scaffolds consisted only of reads obtained from the semi-purified fraction of diseased abalone. Among these 2,160 scaffolds, 70 scaffolds with lengths exceeding 500 bp were selected for further analysis. Among those 70 scaffolds, 15 showed homology to the abalone sequence when subjected to BLASTN analysis; sequences of these 15 scaffolds were not studied further. The remaining 55 scaffolds (with a mean coverage depth of 5,765) were used for a PCR-based epidemiological survey intended to identify scaffolds corresponding to the causative agent.

### RNA shotgun sequencing

Pyrosequencing runs on an RNA library from the semi-purified fraction yielded 2.35 × 10^7^ reads consisting of 2.8 × 10^9^ bases of nucleotide sequence; assembly of these sequences generated 1,198 contigs. Among these contigs, 4 with a length exceeding 500 bp were selected for an RT-PCR-based epidemiological survey intended to identify sequences corresponding to the causative agent.

### PCR- and RT-PCR-based epidemiological surveys

Fifty-five scaffolds from DNA sequencing and 4 contigs from RNA sequencing were screened by PCR and RT-PCR, respectively, using nucleotides extracted from diseased and healthy abalone as samples. In the first screening using pooled DNA samples, 13 primer sets (targeting Scaffolds 1, 4, 5, 7, 8, 9, 11, 15, 38, 53, 65, 66, and 68; mean read-depth 2,828 ± 1,279) generated amplicons of the expected sizes from the two pools of samples from diseased abalone, and not from the two pools of samples from healthy abalone (Supplemental Fig. 1 and Table 1).

**Figure 2:**
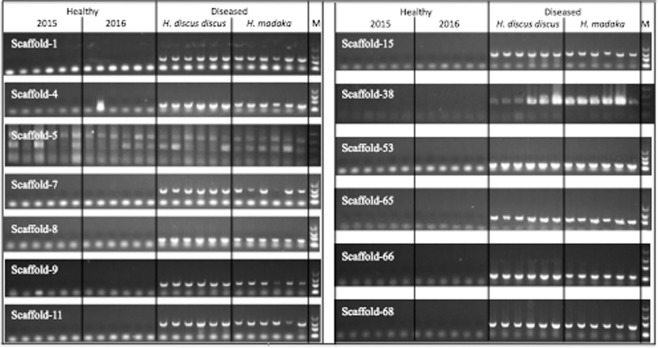
Electrophoresis images of PCR products obtained from individual DNA samples. PCR products from healthy abalone in 2015 and 2016, diseased black abalone *(Haliotis discus discus*), and giant abalone (*Haliotis madaka*) (N = 6 per group) were electrophoresed, stained with GelGreen, and visualized using an LED transilluminator. M indicates the DNA molecular-weight size marker (2,000, 1,000, 500, and 100 bp). Full-length gels are presented in Supplemental Fig. 3.

These 13 primer sets, which yielded amplicons of the expected sizes from the pooled DNA of diseased abalone only, then were tested on the original (unpooled) individual DNA preparations from each abalone. With two exceptions, all the tested primer sets generated amplicons with the expected product sizes from the 12 diseased abalone, but not from the healthy animals (Fig. 2). The first exception was the primer set targeting Scaffold 5, which did not yield specific products from either the diseased or healthy abalone samples. The second exception was the primer set targeting Scaffold 4, which yielded amplicons of the expected size from the 12 diseased samples and from one healthy animal; notably, the sequence of the product generated from the healthy animal did not match the sequences of the amplicons obtained with this primer set from diseased animals nor with the Scaffold 4 sequences obtained from NGS (data not shown). Therefore, the 12 diseased abalone-specific scaffolds (i.e., all but Scaffold 5) were chosen for further analysis.

Equivalent amplification assays were performed on the pooled RNA samples using the four RT-PCR primer sets and RT-PCR. Notably, diseased abalone-specific amplicons were not obtained by RT-PCR with the 4-pooled RNA samples (Supplemental Fig. 1); therefore, RT-PCR tests on the individual RNA samples were not performed.

### Gap closure and genome analysis

Among the 12 scaffolds that were specifically and universally detected in diseased abalone, 10 scaffolds (Scaffolds 1, 7, 8, 9, 11, 15, 53, 65, 66, and 68) were linked into one sequence (Accession No. LC506465) by gap-closing PCR. A draft genome sequence consisting of 1,551,181 bp with a GC content of 31.7% was obtained by the assembly of the NGS sequence and Sanger sequencing of the 9 gap regions. The RAST program^35^ predicted the presence of 159 ORFs in this draft genome. The results of BLASTP (*E-value* < 1e-3) searches of the virus and non-redundant (nr) sequence databases with each of the 159 predicted proteins are summarized in Table 1 and Supplemental Table 3, respectively. The majority of the top hits were obtained against ASFV (44 proteins against proteins encoded by the nr database, 47 proteins against proteins encoded by the virus database). Within the virus database, among the 159 predicted proteins of the novel viruses, 61 proteins show sequence similarity to proteins encoded by other viruses; the top matches were to proteins encoded by members of NCLDVs (58 proteins), primarily to proteins encoded by ASFV (47 proteins) and by ASFV-related species (faustovirus and pacmanvirus; 6 proteins) within the virus database. These BLAST results support the hypothesis that the putative causative agent is closely related to ASFV.

**Table 1:** Top BLASTP hits of AbALV ORFs.

nr database		
	Kingdom	Number of top-hit ORFs
	Virus	47
	*African swine fever virus (ASFV)*	44
	*Pacmanvirus*	2
	*Faustovirus*	1
	Eukaryota	8
	Fungi	3
	Eubacteria	3
	No hit	98
	*Total*	159
**Virus database**		
	**Family**	**Number of top-hit ORFs**
	Asfarviridae (NCLDV)	47
	Asfaviridae? (NCLDV)	6
	Mimiviridae (NCLDV)	2
	Pithoviridae (NCLDV)	1
	Poxvirus (NCLDV)	1
	Phycodnaviridae (NCLDV)	1
	Baculoviridae	1
	Myoviridae	1
	Siphoviridae	1
	No hit	98
	*Total*	159

BLASTP homology search of AbALV ORFs against the nonredundant and viral databases (E-value<1e-3).

Numbers indicate the number of sequences of each kingdom and virus family possessing high homology with the AbALV ORFs.

Asfarviridae: African swine fever virus (ASFV).

Asfaviridae?: faustovirus and pacmanvirus.

Two scaffolds (Scaffolds 4 and 38) were not linked by gap-closing PCR. Thirty-eight ORFs were predicted from Scaffold 4, and one ORF encoded a protein with sequence similarity to a protein encoded by a Planctomycetes bacterium (as assessed by BLASTP against the nr database). Notably, none of these 38 ORFs yielded hits against the virus database. Scaffold 38 was predicted to harbor 4 ORFs, but none of the resulting predicted proteins exhibited similarities by BLASTP against either the nr or virus database.

### Phylogenetic and syntenic analyses

**Figure 3:**
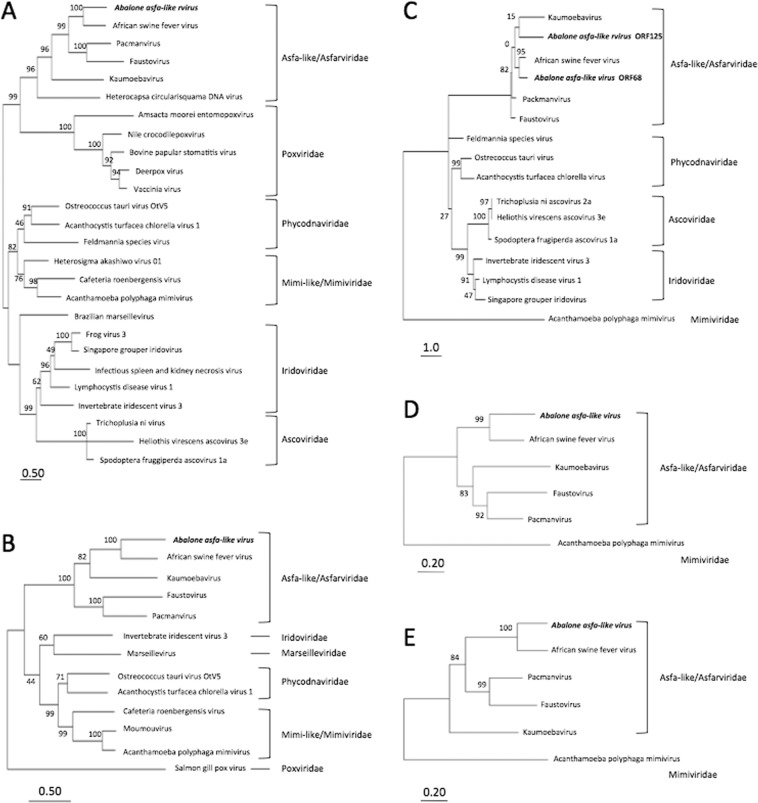
Phylogenetic tree based on the deduced amino acid sequences of abalone asfa-like virus (AbALV)-encoded DNA polymerase (**A**), topoisomerase (**B**), major capsid protein (MCP) (**C**), DNA-directed RNA polymerase 1 (**D**), and DNA-directed RNA polymerase 2 (**E**) sequences compared to homologous proteins in other nucleocytoplasmic DNA viruses. The tree was constructed by the maximum-likelihood method using MEGA7^46^; the numbers at nodes indicate percentages of bootstrap support from 1,000 replicates each. Bar indicates expected amino acid substitutions per site. Two MCP proteins predicted from the AbALV genome were analyzed separately, but full-length MCP protein after splicing was analyzed from faustovirus and kaumoebavirus.

The predicted DNA polymerase B (Fig. 3A), topoisomerase (Fig. 3B), two major capsid proteins (MCPs; encoded by ORF68 and ORF125; Fig. 3C), RNA polymerase 1 (Fig. 3D), and RNA polymerase 2 (Fig. 3E) proteins of the putative causative agent were phylogenetically analyzed by comparison to homologous proteins encoded by NCLDVs. These five proteins sorted with the Asfa-like/Asfarviridae family and were most closely related to the respective proteins encoded by ASFV. For the two putative MCPs, the shorter sequence (encoded by ORF125) clustered with a protein from kaumoebavirus, but with a low bootstrap value (15%).

**Figure 4:**
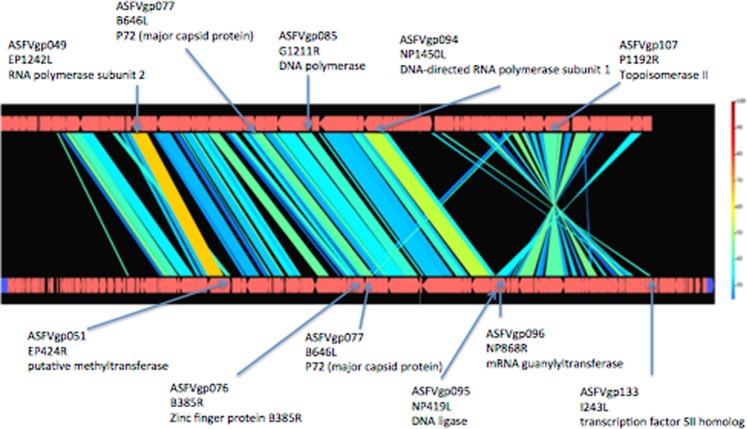
Syntenic analysis of abalone asfa-like virus (AbALV) and African swine fever virus (ASFV). Comparative genomics with AbALV (as obtained in the present study; incomplete genome) is shown at the top and reference genome of ASFV Georgia 2007/1^36^ is shown at the bottom; the figure was generated using GenomeMatcher^47^. Genomic regions with >30% nucleotide identity are joined (top vs. bottom). The color scale on the right-hand side indicates percent protein identity. Black areas with no joining represent regions that lack synteny. Proteins used for phylogenetic analysis (top) and inversion and translocation sites (bottom) are indicated by arrows, and the corresponding ASFV genes are indicated by locus tag, locus, and protein name.

Draft genome alignment of the putative causative agent with the reference genome of ASFV Georgia 2007/1^36^ revealed large syntenic regions and overall conservation of gene order, albeit with inversion and translocation (Fig. 4). The inversion occurred between sites corresponding to locus tag gp096 (NP868R mRNA guanylyltransferase gene) and gp133 (I243L transcription factor S2 homolog gene) of the ASFV genome. The translocation occurred between gp051 (EP424R methyltransferase gene), gp076 (B385R zinc finger protein gene), and gp077 (B646L major capsid protein) of the ASFV genome. The position of the longer MCP-encoding gene (ORF68) was conserved with respect to that of the ASFV genome, but the shorter MCP-encoding sequence (ORF125) was present in the inverted region (Fig. 4 and Supplemental Table 3).

Thus, phylogenetic and syntenic analyses indicated the putative causative is most closely related to ASFV among presently known viruses.

### *In situ* hybridization

**Figure 5:**
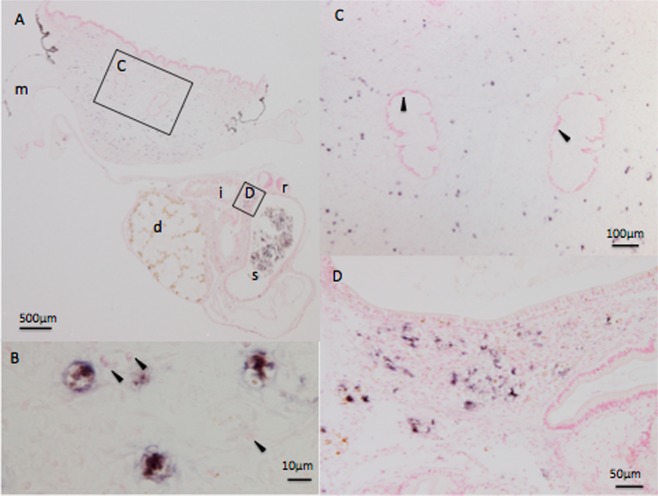
*In situ* hybridization showing the presence of the viral genome in diseased black abalone (*Haliotis discus discus*). Arrows indicate cells positive for staining. (**A**) Cross-section of a diseased abalone. The foot muscle is located on the upper side and the visceral mass is located on the lower side. Squares indicate the magnified fields shown in Panels C and D. The letters d, i, m, r, and s indicate digestive gland, intestine, mantle, stomach, and radula, respectively. (**B**) Higher-magnification image of infected cells. Positive signals were observed in entire cells, including the cytoplasm. Arrowheads indicate the cell nuclei of cells negative for staining. (**C**) Enlarged view around the nerve cord. Note that nerve cells are negative for staining (arrowheads). (**D**) Enlarged view around the digestive tract.

Two DNA probes for *in situ* hybridization (ISH) were generated based on the longer major capsid-encoding gene (ORF68). The two probes yielded similar positive signals only in infected abalone. The results of Probe 1 yielded a slightly stronger signal (Fig. 5). Positive signals for ISH were observed in both of two diseased animals and consisted of signal scattered throughout the muscle (Fig. 5A,C) and connective tissue around the intestinal tract (Fig. 5A,D). No positive signal was seen in the nerve cells (Fig. 5C). Abnormal cell masses characteristic of muscle atrophy were not observed in either of the two diseased abalone assessed by ISH. Positive staining was observed throughout the entire cell, including the cytoplasm (Fig. 5B). No positive hybridization signal was observed in sections of uninfected abalone (data not shown).

## Discussion

Approximately 30 years have passed since abalone amyotrophia was first reported^8^, but the causative agent has remained unknown. In the present study, we successfully identified an ASFV-like virus as the putative causative agent; our analysis combined infection tests with the semi-purified pathogen, NGS analysis, and a PCR-based epidemiological survey. As a result, 12 diseased abalone-specific scaffolds were obtained, all but two of which were arranged into one extended sequence by gap-closing PCR. Eventually, three sequences were left as candidates for the pathogen genome. The longest sequence is the most probable candidate, given that a large number of the proteins predicted from this sequence exhibited sequence similarity to known viral proteins. In contrast, proteins predicted from the two remaining unlinked scaffolds did not exhibit sequence similarity to known viral proteins. The origin of these two unlinked scaffolds remains unclear. We hypothesize that the extended sequence that we recovered originated from the causative agent of abalone amyotrophia, given that this sequence is specific for diseased abalone and is the only sequence (among the 3 scaffolds) encoding homologs of known viral proteins. However, proof of this hypothesis will require isolation and purification of the putative causative agent.

The gap-closed sequence consisted of 1,551,181 bp with a GC content of 31.7% and 159 predicted ORFs. When BLASTP was used to search for sequences with similarity to the 159 predicted proteins encoded by this extended sequence, the majority of the top hits were obtained with ASFV. The phylogeny of five proteins encoded by the gap-closed sequence yielded similar results. With the exception of the short MCP, all the predicted proteins were closest to respective proteins encoded by ASFV. Furthermore, conserved synteny was observed between the partial genome sequence of the abalone virus and ASFV. Therefore, the pathogen is assumed to be a virus that evolved from an ancestor shared with that of ASFV. The virus found from amyotrophic abalone is most closely related to ASFV; we therefore propose provisionally designating this virus as abalone asfa-like virus (AbALV).

The ASFV genome consists of a single-molecule double-stranded DNA with a linear genome length of about 170–193 kb containing between 150 and 167 ORFs, depending on the isolate; the GC content of the ASFV genome is approximately 39%^37^. Because the AbALV genome remains incomplete, comparison of the entire genome to those of other viruses cannot be performed. Nonetheless, the sequenced region of AbALV spans 155 kb, contains 159 ORFs, and exhibits a GC content of 31.7%; all three of these parameters are similar to those of ASFV. Although the overall structures of the ASFV and AbALV genomes are conserved, separate inversion and translocation events were observed in the AbALV genome relative to the ASFV genome, as shown in Fig. 4. The major capsid protein in ASFV is coded as a single ORF, but the MCP of the AbALV genome appears to be encoded as two separate genes. In the ASFV/ASFV-like virus lineage, MCP is encoded as a single ORF in ASFV and pacmanvirus, but it is encoded by multiple loci in both kaumoebavirus^5^ and faustoviruses^38^. Thus, the splitting of MCP-encoding sequences into 2 genes in AbALV resembles the case in kaumoebavirus and faustoviruses. The virological or evolutionary implications of these AbALV genome characteristics are unknown.

ASFV is a large, enveloped virus with icosahedral morphology and an average diameter of 200 nm^37^. The ASFV capsid was reported to have side-to-side dimensions of 172–191 nm^39^. The diameter of the amyotrophia-causing virus was estimated to be 100–220 nm by filtration experiments^9,16^; therefore, the inferred size of AbALV virions would be consistent with the characteristics of ASFV. ASFV is a member of the NCLDV superfamily and replicates primarily in the cytoplasm of macrophages^4^. ISH in the present study yielded positive signals throughout the entire cytoplasm of infected cells, another property that is consistent with that of ASFV. Among viruses reported by electron microscopic (EM) observation of amyotrophic abalone^9,28,29^, that observed by Otsu^29^ was the most similar to ASFV. Notably, Otsu observed virus-like particles with a diameter of approximately 100 nm in the cytoplasm of cells near the nerve trunk of diseased black abalone. However, no information is available that links the virus observed by Otsu to AbALV. While we also performed transmission EM (TEM) evaluation of diseased black abalone, we did not observe virus-like particles (data not shown). Difficulty in observing the causative virus by EM likely reflects dispersion of infected cells throughout the connective tissue, as shown by ISH. Additionally, we suspect that there are large individual differences in the amount of virus in the tissue. Specifically, the titer of the AbALV genome (a parameter that was measured to select samples for ISH) in the muscle of 20 diseased animals ranged from 2.3 × 10^1^ to 1.9 × 10^5^ copies/mg wet tissue weight as judged by AbALV-targeted quantitative PCR. Negative staining and TEM observation of the semi-purified fraction used for NGS also was attempted, but spheroids of various sizes were found in the fractions prepared from both healthy and diseased abalone, precluding identification of the virus by EM (Supplemental data 1). Further characterization of this virus will require observation of viral morphology by EM.

In ISH images, positive signals were scattered throughout cells of the foot muscle and connective tissues without demonstrating accumulation in specific tissues. Muscular atrophy is characterized by the formation of abnormal cell masses around the ganglion, but no positive signal was obtained in the nerve cells. Unfortunately, abnormal cell masses were not formed in the two diseased abalone analyzed, and the relationship between the virus-infected cells and abnormal cell masses could not be demonstrated in our analysis. Given the lack of information about abalone cells, the target cells could not be identified from the ISH images. Based on the form and size of cells that exhibited positive staining by ISH, the infected cells appear not to be hemocytes or muscle cells. Identification of virus target cells will be important for understanding how viral infections yield pathologies such as abnormal cell mass formation, incisions on the shell, and death.

ASFV primarily infects monocytes and macrophages^40,41^, but the Asfa-like viruses faustovirus^4^, kaumoebavirus^5^, and pacmanvirus^6^ have been isolated using amoeba as the host. We attempted to isolate AbALV using black abalone hemocytes and amoeba (*Acanthamoeba polyphaga* and *A. castellanii*) as host cells, but no cytopathic effect (CPE) was observed, nor was virus proliferation detected by quantitative PCR (data not shown). Failure to isolate the causative virus using a primary culture of abalone hemocytes from the amyotrophic abalone had been reported previously^28^. Based on our ISH results, we infer that AbALV likely infects a cell type other than hemocytes. Identification of viral target cells also will be necessary for isolation of the virus using host cells, as well as to understand the pathological nature of AbALV.

ASFV causes an acute, high-mortality disease in domestic pigs. However, in its natural hosts the warthog (*Phacochoerus africanus*)^42^, bushpig (*Potamochoerus* spp.)^43^, and soft tick (*Ornithodoros moubata*)^2^, ASFV has low pathogenicity while causing persistent infections. In its natural state, ASFV is maintained in the sylvatic cycle in the warthog, bushpig, and soft tick without inducing clinical signs of disease^4^. It is not clear which organisms are the evolutionally original host of ASFV, but the present work indicates that a virus similar to ASFV was detected in abalone, which belongs to the protostome clade similar to ticks. This observation supports the idea that ASFV originally was a virus that used an arthropod (e.g., ticks) as a host, but not mammals (e.g., pigs), before evolution to gain the ability to infect and propagate in the warthog, a tick host. Analysis of the virological properties of AbALV is expected to facilitate protecting abalone from disease while also bringing new insight into ASFV pathology and evolution.

## Materials and Methods

### Animals

**Figure 6:**
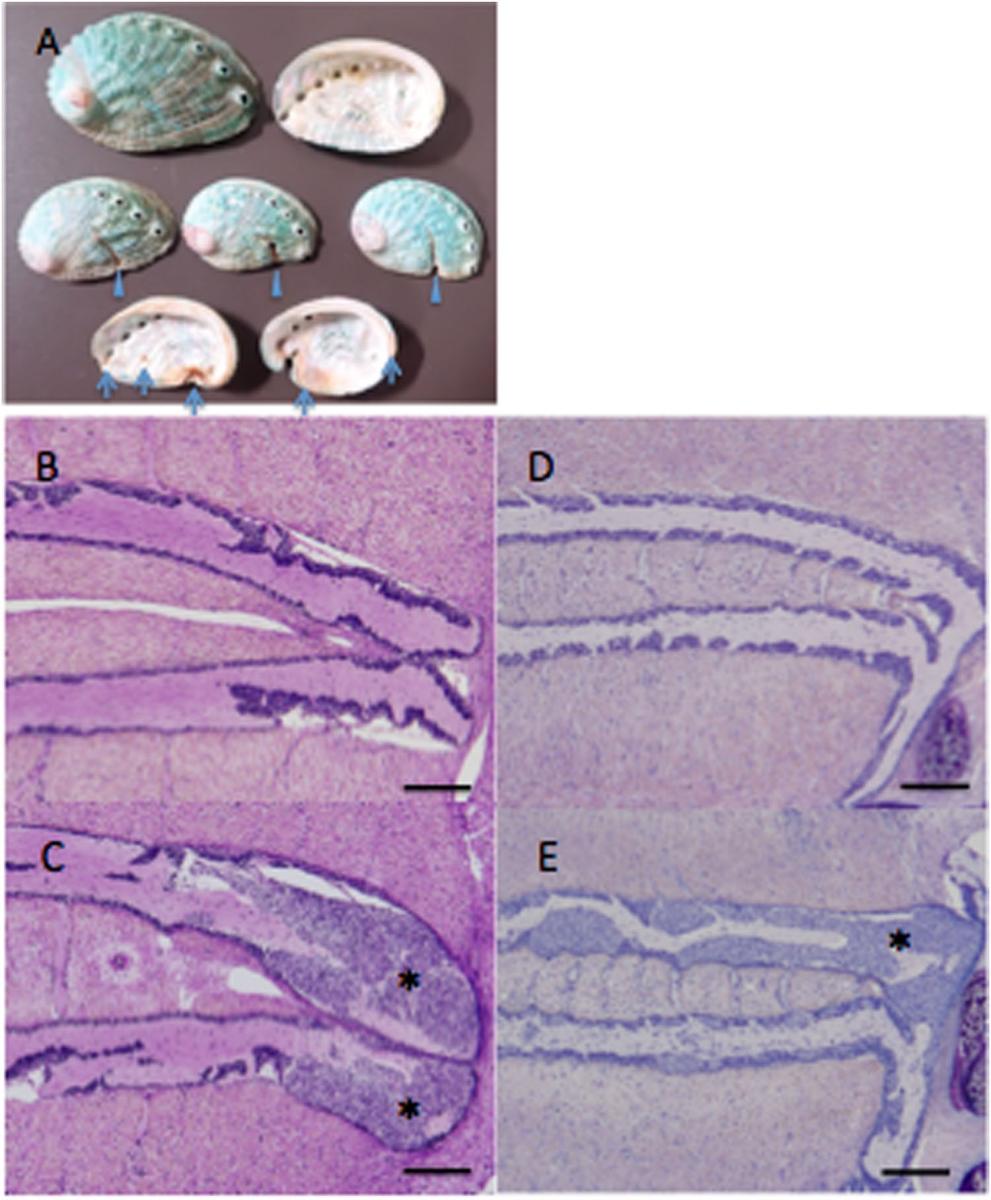
External appearance and histopathological characteristics of abalone with amyotrophia. (**A**) Photograph of diseased black abalone shells with symptomatic incisions on the front margin of the shells (arrowheads) and brown pigmentation inside the shell (arrows). Histological observation of healthy (**B**) and diseased (**C**) black abalone (*Haliotis discus discus*) and healthy (**D**) and diseased (**E**) giant abalone (*Haliotis madaka*). Abnormal cell masses (*) are observed around the ganglia in both species. Black bars indicate 200 µm.

Diseased black abalone (*H. discus discus*) and giant abalone (*H*. *madaka*) with high mortality were obtained respectively from the city of Omaezaki, Shizuoka Prefecture, on June 30, 2015, and from the town of Ikata, Ehime Prefecture, on June 9, 2016. The diseased black abalone exhibited incisions on the front margin of the shells and brown pigmentations on the inner surface of the shell (Fig. 6A); these symptoms were not observed in diseased giant abalone (data not shown). Both diseased abalone species exhibited abnormal cell masses when subjected to histopathological examination (Fig. 6B). Healthy black abalone were obtained from the city of Owase, Mie Prefecture, on September 17, 2015 and April 18, 2016. No external appearance or histological abnormalities were found in the healthy abalone. All animal experiments were approved by the Japan Fisheries Research and Education Agency Institutional Animal Care and Use Committee and performed in accordance with relevant guidelines and regulations under license 27003.

### Histological observation

Abalone was preserved in Davidson’s solution^44^ and processed for routine paraffin histology. Paraffin-embedded tissue blocks were sectioned at 3-µm thicknesses, stained with May Grunwald’s Giemsa, and observed with a BX51 microscope (Olympus).

### Semi-purification of causative agent

Approximately 0.5 g of soft-body tissues, excluding the midgut, were recovered from each of 6 healthy black abalone from Owase in 2015 and from each of 6 diseased black abalone from Omaezaki at the indicated times. Tissues were minced with a razor, combined with 10 volumes of autoclaved seawater, and crushed with a Potter-type glass homogenizer. The crushed samples were centrifuged at 15,000 × *g* for 10 min at 4 °C, and the supernatants were passed through a 0.22-μm syringe filter (Millipore). Aliquots of the filtrates were stored at −80 °C for use as the infectious sources for the positive (diseased) and negative (healthy) control groups. The balance of the filtrate from diseased abalone was diluted with autoclaved seawater to 25 mL and recombinant DNase I and RNase A (TAKARA Bio) were added to final concentrations of 100 U/mL and 200 µg/mL, respectively. Immediately following the addition of the nucleases, samples were centrifuged at 150,000 × *g* for 1 h at 25 °C to sediment the particles; digestion of free nucleic acids proceeded during the ultracentrifugation. The resulting supernatant was decanted and 12 mL of autoclaved seawater was added to each tube; the tubes then were incubated for 2 h on ice to resuspend the pellet. Again, DNase I (100 U/mL) and RNase A (200 µg/mL) were added to each tube, and the samples were subjected to another round of ultracentrifugation (150,000 × *g*, 1 h, 25 °C). The resulting supernatant was decanted and 12 mL of autoclaved seawater was added to each tube; the tubes were incubated for 12 h on ice to resuspend the pellet before being subjected to another round of ultracentrifugation to remove remaining nuclease. The resulting supernatant was decanted and 1 mL of autoclaved seawater was added to each tube; the tubes were incubated for 2 h on ice to resuspend the pellet. The samples then were centrifuged at 15,000 × *g* for 10 min at 4 °C, and the resulting supernatant (designated as the semi-purified sample) was aliquoted at 200 μL/tube and stored at −80 °C until analysis.

### Infection test

Healthy black abalone (body length: range 22–27 mm; mean, 23.9 ± 1.7 mm) were used as the recipient of the infection test. The semi-purified sample was diluted 2-fold with autoclaved seawater and injected into the foot muscles of 40 healthy black abalone (20 μL/animal) with a 30-gauge needle. Filtrates prepared as described above (i.e., subjected solely to 0.22-µm filtration) from diseased and healthy abalone were used as inocula for the positive and negative control groups, respectively, and injected into 20 healthy abalone/group using the same procedure as for the semi-purified sample. The amount of virus was estimated to be 7.5 × 10^4^ and 7.3 × 10^2^ copy/animal in inocula for positive control and semi-purified fraction-infected group respectively, based on absolute quantification using quantitative PCR (methods are described below). For the semi-purified fraction-infected group (N = 40), abalone were divided into 2 groups (N = 20 each) and reared in separate 56-L tanks. The two control groups were reared in separate tanks. All groups were reared in running seawater maintained at 19 ± 2 °C and fed with a commercial pelleted diet once per week. Three surviving animals from each group were recovered for histological observation at the end of the infection test.

### Sequencing and data processing

RNA and DNA were isolated from the semi-purified samples using NucleoSpin RNA NX and NucleoSpin tissue XS (TAKARA Bio), respectively. DNA also was purified from the muscle of a healthy abalone. RNA-seq libraries were prepared from RNA sample using the ScriptSeq v2 RNA-Seq Library Preparation kit (Illumina), and sequenced on the NextSeq 500 sequencer (Illumina) with single-end reads of 151 bp according to the manufacturer’s protocol. DNA from the semi-purified fraction was amplified using Illustra Single Cell GenomiPhi DNA Amplification Kits (GE Healthcare). The libraries were constructed from amplified semi-purified fraction DNA and healthy abalone DNA using TruSeq Nano DNA LT Sample Prep Kits and sequenced on the NextSeq 500 sequencer with paired-end reads of 2 × 151 bp according to the manufacturer’s protocol.

RNA sequencing data were assembled de novo with the CLC Genomics Workbench 7.0.3 (Qiagen) under default parameters, and contigs longer than 500 bp were selected for further analysis. Processing of the DNA sequencing data was conducted as follows. The sequencing data were filtered and trimmed using Trimmomatic^45^ v. 0.3.6 (CROP: 145; LEADING: 30; TRAILING: 20; SLIDINGWINDOW: 4:20; MINLEN: 50). Trimmed reads obtained from the semi-purified fraction library and healthy abalone library were mixed and de novo assembled with Platanus 1.2.4. (http://platanus.bio.titech.ac.jp) under default parameters to generate scaffolds. Scaffolds that formed only from reads obtained from the semi-purified fraction library were extracted. Scaffolds longer than 500 bp were selected and homology-searched against the NCBI nr database; sequences classified as abalone sequences were removed. Residual scaffolds were subjected to further analysis.

### PCR- and RT-PCR-based epidemiological surveys

Using primer3 software (http://bioinfo.ut.ee/primer3-0.4.0/), primers were designed to target the 55 diseased abalone-specific scaffolds that were obtained from DNA sequencing, and 4 contigs longer than 500 bp that were obtained from RNA sequencing (Supplemental Table 1). For scaffolds against which primers could not be designed with primer3, primers were designed manually. DNA and RNA of diseased abalone were obtained from the muscle of diseased animals obtained from Omaezaki (N = 6) and Ikata (N = 6), and from the muscles of healthy animals obtained from Owase in 2015 (N = 6) and 2016 (N = 6). DNA and RNA were extracted with QIAamp DNA Mini kits (Qiagen) or TRIzol reagent (Thermo Fisher Scientific), respectively. PCR assays were performed using KOD FX (TOYOBO) in 20-μL reactions containing 1 μL of sample DNA. Pooled DNA samples (consisting of equal volumes of DNA from each of the 6 abalone per group) were used as PCR templates for testing all 55 primer sets. Individual DNA samples were used as PCR templates to test primer sets for which diseased abalone-specific amplification was seen by PCR of pooled DNA samples. The PCR amplification program consisted of 35 cycles of denaturation at 98 °C for 10 s, annealing at 65 °C for 30 s, and extension at 68 °C for 90 s. For RT-PCR, reverse transcription (RT) was performed using a mixture of random nonamers and oligo-(dT) primer along with 1 μg total RNA; RT was performed using the ReverTra Ace kit (TOYOBO), and the subsequent PCR was carried out as above using 1 μL of the reverse transcription product as a template. Pooled RNA samples (consisting of equal volumes of RNA from each of the 6 abalone per group) were used as RT templates for testing 4 primer sets. PCR products were separated on 1.5% agarose gels, and the gels were stained with GelGreen (Wako) and visualized using an LED transilluminator.

### Gap closure and sequencing

PCR primers (Supplemental Table 2) were manually designed to close gaps between 12 scaffolds, which had been selected by the survey described above. PCR was performed using the KOD FX kit with a pooled DNA sample of diseased abalone obtained from Omaezaki. Amplification was as described above, except that the extension step was elongated to 10 min. PCR products were analyzed using agarose gel electrophoresis, and DNA from positive reactions were purified using the Wizard SV Gel and PCR Clean-Up System (Promega). Direct sequencing was performed using the ABI PRISM BigDye Terminator v3.1 Cycle Sequencing Kit (Applied Biosystems) and the ABI PRISM 377 DNA sequencer (Thermo Fisher Scientific).

### Genome annotation and phylogenetic and syntenic analyses

The RAST program^35^ was used for gene prediction of the genome sequence of the putative causative agent. BLASTP searches against the nr and virus sequence databases (as of September 13, 2019) were performed with each of the predicted proteins.

Maximum-likelihood phylogenetic trees were constructed using protein sequences predicted from the genes encoding DNA polymerase B, topoisomerase, MCPs, and RNA polymerases 1 and 2. The protein sequences included those encoded by the type species for each NCLDV, related species, and the respective sequence obtained in the present study. Trees were created using 1,000 bootstrap replicates of the sequence alignments in MEGA 7^46^.

The genome sequence of the putative causative agent was compared with the reference genome of ASFV Georgia 2007/1^36^ and visualized for syntenic comparison using GenomeMatcher^47^.

### Quantitative PCR

For quantitative PCR (qPCR), specific primers were designed (Q-ASFV-like-F: 5′-cccggagcgacctacagaa-′3, Q-ASFV-like-R: 5′-gcattccgacagcatcacag-′3) that generate 127-bp amplicons within the MCP gene (GenBank Accession No. BBO54023) of AbALV. The complete MCP gene of AbALV was PCR amplified from diseased abalone sample from Omaezaki using specific primer sets (p72-F: 5′-atggcggcaggaggacccttcattttgataacaaac-′3, p72-R: 5′-ttatgcagcatatcgcaagatagctgatccgtcggtg-′3) and inserted into the pCR2.1 plasmid vector to generate standards for quantification.

qPCR assays were performed using the Stratagene Mx3000p qPCR system and companion software, MxPro (Stratagene). KOD SYBR qPCR Mix (Toyobo) was used for the reactions according to the manufacturer’s protocol. Briefly, qPCR was performed in 20-μL reactions containing 10 μL of kit-supplied buffer (2×), 50 pmol of primers, 0.1 μL of ROX, and 1 μL of sample DNA. qPCR assays were performed under the following cycling conditions: activation of Hot-Start DNA polymerase at 95 °C for 10 min, followed by 35 amplification cycles of 95 °C for 10 s, 60 °C for 10 s, and 68 °C for 30 s. Specificity was evaluated based on melting curve analyses conducted after amplification, as follows: 95 °C for 1 min, 55 °C for 1 min, followed by an increase in temperature of 0.5 °C every 10 s from 55 °C to 95 °C. The melting curve data were plotted according to the manufacturer’s instructions (Stratagene). Standard curves were constructed using a plasmid vector containing the MCP fragment. Plasmid vector copy number was determined based on molecular weight. Gene copy number was calculated per microliter for inoculum or per milligram of tissue.

### *In situ* hybridization

An infection test was conducted to generate diseased black abalone for ISH. Twenty diseased abalone were injected (as described above) with diseased black abalone homogenate; at 28 dpi, tissue samples from each animal were screened for titer of the putative AbALV agent using qPCR. Two animals with high levels of AbALV genomic DNA infection titers were selected and used as sources of tissue samples for ISH. ISH was performed under contract with Genostaff Co., Ltd. The longer MCP-encoding gene (ORF68) of AbALV was cloned into the pGEM-T Easy vector (Promega) and used for generation of digoxigenin (DIG)-labeled probes via the DIG Probe Synthesis Kit (Roche Diagnostics) according to the manufacturer’s instructions. The resulting probes corresponded to nucleotide positions 385–942 (Probe-1, 558 bp) and 1440–1553 (Probe-2, 414 bp) of ORF68. Tissue samples from four abalone total (two diseased animals and two healthy animals) were fixed with G-Fix (Genostaff), embedded in paraffin, and sectioned at 6-µm thicknesses. ISH was performed with the ISH Reagent Kit (Genostaff) according to the manufacturer’s instructions. Hybridization was performed separately with either of the two probes at concentrations of 150 ng/mL in G-Hybo-L (Genostaff) for 16 h at 40 °C. Following hybridization, the sections were washed with 50% formamide in 2xG-Wash (Genostaff) and then with Tris-buffered saline containing 0.1% Tween 20 (TBST). The washed sections were incubated with alkaline phosphatase-conjugated anti-DIG antibody (Roche Diagnostics) diluted 1:2000 with 50x G-Block (Genostaff) in TBST for 1 h at room temperature. Color development was performed with NBT/BCIP solution (Sigma-Aldrich), and sections then were counterstained with Kernechtrot stain solution.

### Statistical analysis

Where applicable, data are presented as mean ± standard deviation (SD). Cumulative mortality was analyzed statistically with Fisher’s exact tests. A *p* value of <0.05 was considered significant.

## Supplementary information

Supplementary information

Supplementary information2
