# Author Correction: A novel Asfarvirus-like virus identified as a potential cause of mass mortality of abalone

**DOI:** 10.1038/s41598-020-65515-x

**Published:** 2020-05-15

**Authors:** Tomomasa Matsuyama, Tomokazu Takano, Issei Nishiki, Atushi Fujiwara, Ikunari Kiryu, Mari Iinada, Takamitsu Sakai, Sachiko Terashima, Yuta Matsuura, Kiyoshi Isowa, Chihaya Nakayasu

**Affiliations:** 1 National Research Institute of Aquaculture, Japan Fisheries Research and Education Agency, Research Center for Fish Diseases, Minami-Ise, Mie 516-0193 Japan; 2 National Research Institute of Fisheries Science, Japan Fisheries Research and Education Agency, Research Center for Bioinformatics and Biosciences, Yokohama, Kanagawa 2368648 Japan; 3 Japan Fisheries Research and Education Agency, Minato Mirai, Nishi-ku, Yokohama, Kanagawa 2206115 Japan; 4 National Research Institute of Aquaculture, Japan Fisheries Research and Education Agency, Diagnosis and Training Center for Fish Diseases, Minami-Ise, Mie 516-0193 Japan; 5 Mie Prefectural Sea Farming Center, Owase, Japan

Correction to: *Scientific Reports* 10.1038/s41598-020-61492-3, published online 12 March 2020

This Article contains typographical errors.

In the Results under subheading ‘Gap closure and genome analysis’,

“A draft genome sequence consisting of 1,551,181 bp with a GC content of 31.7% was obtained by the assembly of the NGS sequence and Sanger sequencing of the 9 gap regions.”

should read:

“A draft genome sequence consisting of 155,181 bp with a GC content of 31.7% was obtained by the assembly of the NGS sequence and Sanger sequencing of the 9 gap regions.”

In the Discussion,

“The gap-closed sequence consisted of 1,551,181 bp with a GC content of 31.7% and 159 predicted ORFs.”

should read:

“The gap-closed sequence consisted of 155,181 bp with a GC content of 31.7% and 159 predicted ORFs.”

