# Supplementary material for: A novel Asfarvirus-like virus identified as a potential cause of mass mortality of abalone
Source: Sci Rep. 2020 Mar 12;10:4620. doi: 10.1038/s41598-020-61492-3 (PMC7067878; doi:10.1038/s41598-020-61492-3)
Supplement: Supplementary file 2 — Supplementary information2 [file 41598_2020_61492_MOESM2_ESM.pdf]

# A novel Asfarvirus-like virus identified as a potential cause of mass mortality of abalone

Tomomasa Matsuyama*[1], Tomokazu Takano[1], Issei Nishiki[2], Atushi Fujiwara[3], Ikunari Kiryu[4], Mari Inada[4], Takamitsu Sakai[1], Sachiko Terashima[1], Yuta Matsuura[1], Kiyoshi Isowa[5] and Chihaya Nakayasu[1]

1 National Research Institute of Aquaculture, Japan Fisheries Research and Education Agency, Research Center for Fish Diseases, Minami-Ise, Mie 516-0193, Japan
2 National Research Institute of Fisheries Science, Japan Fisheries Research and Education Agency, Research Center for Bioinformatics and Biosciences, Yokohama, Kanagawa 236-8648, Japan
3 Japan Fisheries Research and Education Agency, Minato Mirai, Nishi-ku, Yokohama, Kanagawa 220-6115, Japan
4 National Research Institute of Aquaculture, Japan Fisheries Research and Education Agency, Diagnosis and Training Center for Fish Diseases, Minami-Ise, Mie 516-0193, Japan
5 Mie Prefectural Sea Farming Center, Shima, Japan

*Corresponding author:
Tomomas Matsuyama, Ph. D.

*Japan Fisheries Research and Education Agency,*
*National Research Institute of Aquaculture,*
*Research Center for Fish Diseases,*
*Minami-Ise, Mie 516-0193, Japan*
Tel.: +81-599-66-1830
Fax: +81-599-66-1962
E-mail: matsuym@fra.affrc.go.jp

Supplemental data 1. Negative staining and TEM observation of the semi-purified fraction.

Semi-purified fractions were prepared from cryopreserved (−80 °C) healthy and diseased black abalone collected from Owase and Omaezaki, respectively, in 2015. Approximately 0.1 g of soft-body tissues, excluding the midgut, were recovered from each abalone. Tissues were minced with a razor, combined with 10 volumes of autoclaved seawater, and crushed with a Potter-type glass homogenizer. The crushed samples were centrifuged at 15,000 × *g* for 10 min at 4 °C, and the supernatants were passed through a 0.22-μm syringe filter (Millipore). The filtrates were diluted with autoclaved seawater and centrifuged at 150,000 × *g* for 1 h at 4 °C to sediment the particles. Supernatants were decanted and the resulting pellets were resuspended in a small amount of autoclaved seawater. Resuspended samples were divided for negative staining and TEM observation.

For negative staining observation, samples were absorbed to formvar film-coated grids and stained with 2% phosphor tungstic acid solution. The grids were observed using a transmission electron microscopy (JEOL1400 plus, Jeol).

For TEM observation, resuspended samples were sandwiched between copper disks and frozen in liquid propane at −175 °C. Frozen samples were submerged in 2% glutaraldehyde, 1% tannic acid in ethanol, and 2% distilled water at −80 °C for 2 days. The samples were dehydrated through ethanol and infiltrated with propylene oxide (PO) and were put into a 70:30 mixture of PO and resin (Quetol-812, Nisshin EM Co.) for 1 h. The samples were transferred to fresh 100% resin and polymerized. Thin sections (70

nm) were prepared and stained with 2% uranyl acetate and examined using the JEOL1400 plus transmission electron microscope.

Fig. S2 shows an example of electron microscopic (EM) observation. In images obtained from both analyses, spheroids of various sizes were found in the fractions prepared from both healthy and diseased abalone. AbALV was difficult to identify from observed particle morphology.

Electrophoresis images of PCR products generated by primers designed with primer3

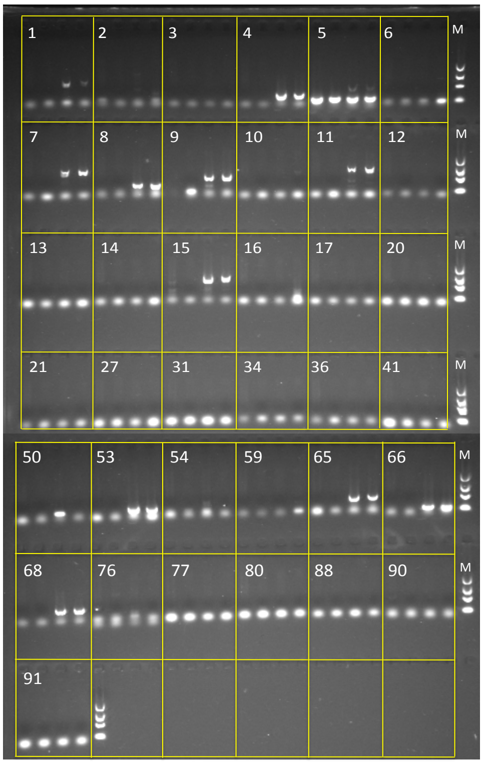


Electrophoresis images of PCR products generated by manually designed primers

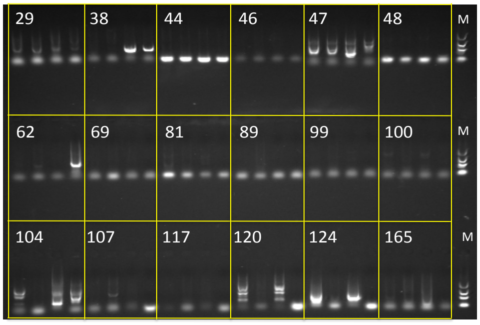


Electrophoresis images of RT-PCR products

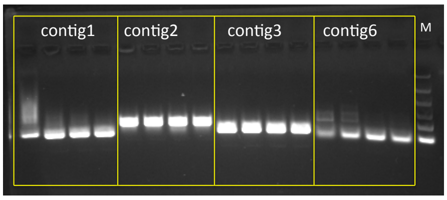


# Supplemental Fig 1

Supplemental Figure 1. Electrophoresis images of PCR and RT-PCR products for pooled DNA/RNA samples. The squares and numbers indicate electrophoresis images of target genes corresponding to each number. PCR products from healthy abalone isolated in 2015 and 2016, diseased black abalone (*Haliotis discus discus*), and diseased giant abalone (*Haliotis madaka*) (N=6 per group) are shown. M indicates the DNA molecular-weight size marker (2,000, 1,000, 500, and 100 bp for PCR; 600, 500, 400, 300, 200, and 100 bp for RT-PCR).

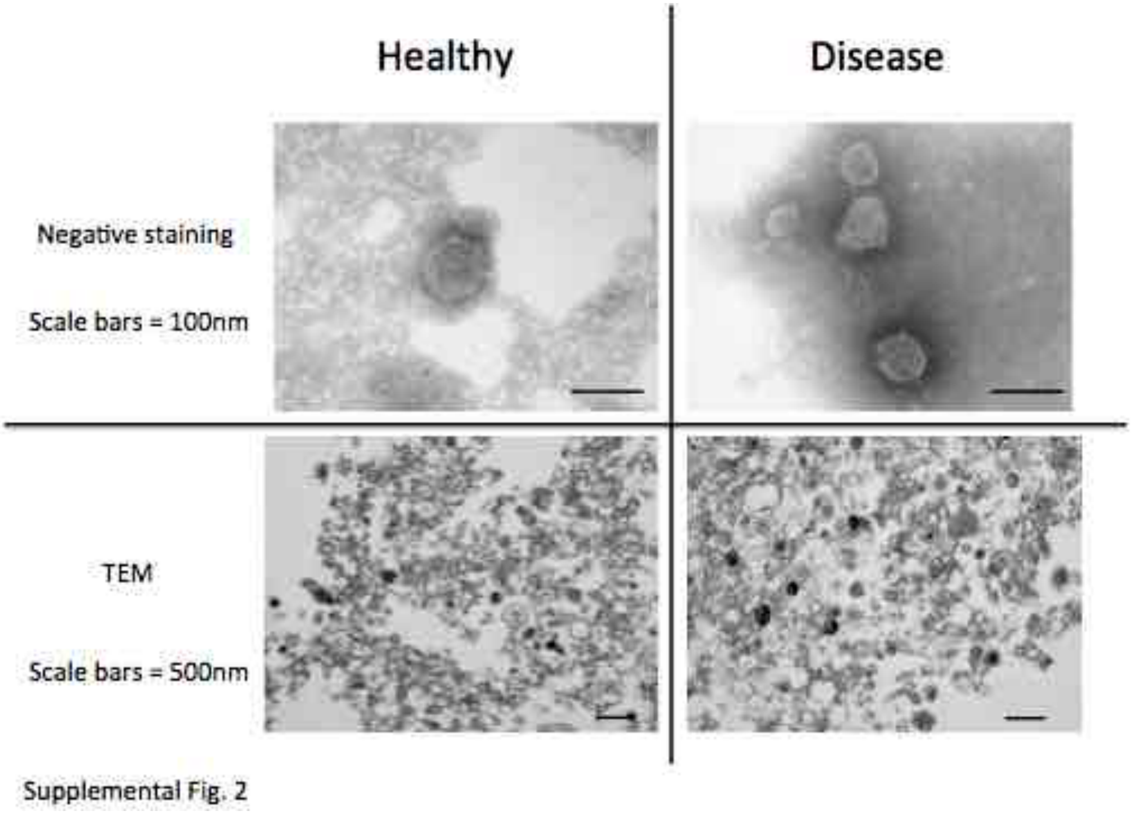


Supplemental Figure 2.

Example of electron microscopic (EM) observation. In images obtained from negative staining and TEM analyses, spheroids of various sizes were found in the fractions prepared from both healthy and diseased abalone.

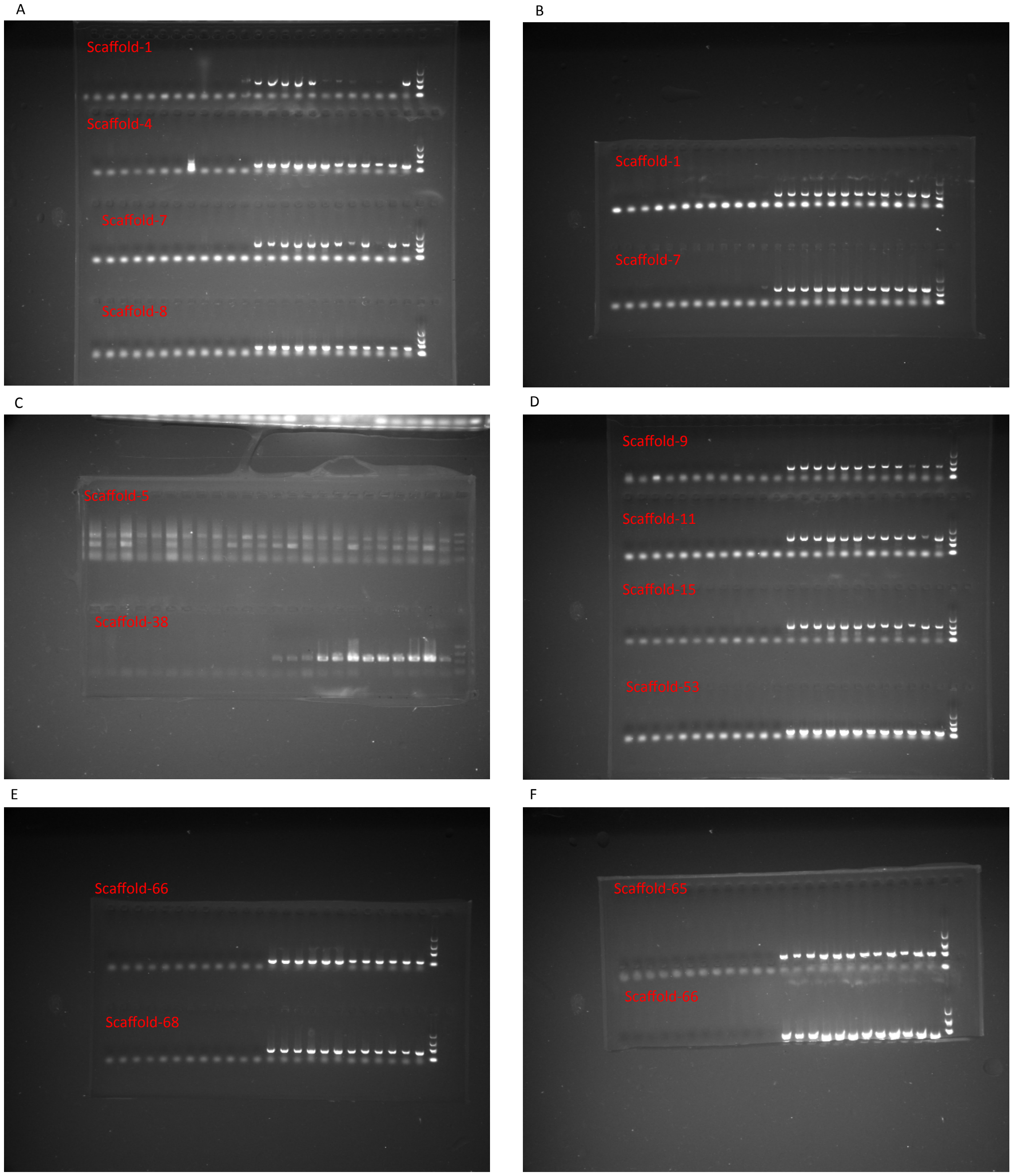


Supplemental Figure 3. Full-length electrophoresis images for analyses described in Figure 2.

(A) Electrophoresis images for Scaffolds 1, 4, 7, and 8. Because Scaffold 1 had many false-negative results among diseased abalone, PCR and electrophoresis were repeated,

and the results from the repeated test (B) were described in the main text. (B) Electrophoresis images for Scaffolds 1 and 7. Scaffold 7 electrophoresis images show a weak positive signal from a healthy abalone sample in the 12th well. Thus, results from (A) were described in the main text. (C) Electrophoresis images for Scaffolds 5 and 38. (D) Electrophoresis images for Scaffolds 9, 11, 15, and 53. (E) Electrophoresis images for Scaffolds 66 and 68. (F) Electrophoresis images for Scaffolds 65 and 66. Because the Scaffold 66 PCR product migrated too far, the image shown in (E) was used in the main text. PCR products from healthy abalone in 2015 and 2016, diseased black abalone *(Haliotis discus discus*), and giant abalone (*Haliotis madaka*) (N=6 per group) were electrophoresed, stained with GelGreen, and visualized using an LED transilluminator. The rightmost lane is the DNA molecular-weight size marker (2,000, 1,000, 500, and 100 bp).

Supplemental Table 1. Primers for PCR- and RT-PCR-based screening of sequences possibly derived from putative causative agent, and results of PCR and RT-PCR for pooled DNA samples

| Scaffolds | Scaffold length (bp) | Reads mapped | Mean coverage | F-primer | sequence(5'-3') | R-primer | Sequence (5'-3') | Expected product size (bp) | Results of PCR and RT-PCR of pooled DNA sample |
|---|---|---|---|---|---|---|---|---|---|
| ***Primers designed by Primer3*** | | | | | | | | | |
| scaffold_0001 | 49,616 | 715,710 | 1,983 | F-sc_0001 | GCGAAATTCCGATTTTGGGCTGTTCGCCCG | R-sc_0001 | TCAAAATGAAGGGTCCTCCTGCCGCCATCG | 795 | D |
| scaffold_0002 | 39,570 | 1,726,916 | 6,135 | F-sc_0002 | AGGCCCGCGATCCCGCCGTGGT | R-sc_0002 | CCGCCGCCTTGGCCGCTTGGGGT | 1259 | N |
| scaffold_0003 | 30,917 | 733,072 | 3,199 | F-sc_0003 | TGCCCTTAATTGGGGAGAGATGATCCGACACT | R-sc_0003 | TCCCCGTTTAGGTTCAGAAGAGCCGTCG | 523 | N |
| scaffold_0004 | 19,721 | 349,693 | 2,412 | F-sc_0004 | CGGGCCCGCGTACCGGAATCGGC | R-sc_0004 | TCCGGCCACCCAAAGTCTGCCCA | 341 | D |
| scaffold_0005 | 18,328 | 316,967 | 2,355 | F-sc_0005 | AGTGGGGAAGGGGTGGGGGCA | R-sc_0005 | ACCAGTGAAGGTCAGGCCCGCCGC | 669 | D |
| scaffold_0006 | 15,747 | 425,616 | 3,679 | F-sc_0006 | TCGTCTATTCCAAACCCTGCCCATTGATCTCCT | R-sc_0006 | ACCCCAGGAGCTTCAGGTACTACAGCGGCA | 538 | N |
| scaffold_0007 | 14,418 | 288,679 | 2,748 | F-sc_0007 | ACGGGCCAACCGATGGACGGCA | R-sc_0007 | GCGGCGGCGGGCCACCAACT | 990 | D |
| scaffold_0008 | 9,744 | 109,705 | 1,541 | F-sc_0008 | TCTGTGTCAAATGAGGGGCGTGGAGGCATT | R-sc_0008 | ATTGGTGTTATGGGGCCGGCCGACTTCTTT | 343 | D |
| scaffold_0009 | 8,540 | 126,524 | 2,030 | F-sc_0009 | TCTTGGTGCGGGTGGCGGCGA | R-sc_0009 | TGGGGGCCGCCCTTCGAATTCCC | 711 | D |
| scaffold_0010 | 7,990 | 111,815 | 1,935 | F-sc_0010 | ACAGGGTGGAGCAGGAACTGGTGCTGA | R-sc_0010 | AGACCCTGAAAGGTTCCTCCCCCAACAGT | 836 | N |
| scaffold_0011 | 7,378 | 148,766 | 2,768 | F-sc_0011 | CCCTTCCCTGGTGCTTCAGAATGGCGCA | R-sc_0011 | TGCAGACCCCGAAATTGAAGCGGCGGT | 1172 | D |
| scaffold_0012 | 6,695 | 68,979 | 1,422 | F-sc_0012 | AGGAGCCATGGCACTTGTACAACAACAACTCC | R-sc_0012 | ACTGCCCTTGTTGGTGAAATAAACACCGGTTCA | 900 | N |
| scaffold_0013 | 6,183 | 61,861 | 1,384 | F-sc_0013 | AACACCAGCACCAGCTGCAGCAACAG | R-sc_0013 | TGATGGCTTTGCTGTGGTAAGGGAGCG | 949 | N |
| scaffold_0014 | 5,566 | 166,266 | 4,114 | F-sc_0014 | ACCCCACCACTATCGGTGAGGACGGA | R-sc_0014 | ACACCACCCGGACTTGGAGCAGGAA | 607 | N |
| scaffold_0015 | 5,385 | 71,380 | 1,810 | F-sc_0015 | CCCCATTGTGCCCATGTTTCTACTGCACCA | R-sc_0015 | TGTGGCTTTTACAATTGCCGAGGCCGAACAA | 1134 | D |
| scaffold_0016 | 4,538 | 52,969 | 1,592 | F-sc_0016 | TAGCCTTGCTGGTGCTGTGCACTCTGCATT | R-sc_0016 | TAACTGCGTTGTTGCGCCCTTAGTGGTGGT | 1494 | N |
| scaffold_0017 | 4,005 | 219,083 | 7,536 | F-sc_0017 | GGAACAGCCCCAACCCCTCCTCTGTCC | R-sc_0017 | TCTGCATCTTTAGGTGGAGTTCGTCCGTTGGT | 1190 | N |
| scaffold_0020 | 3,586 | 140,370 | 5,376 | F-sc_0020 | AACGCCGAGCCAACGGACGGAAAACAGTAG | R-sc_0020 | TTTCCTCCTTGCGCACTTCCACCTCCTTGG | 474 | N |
| scaffold_0021 | 2,853 | 57,456 | 2,662 | F-sc_0021 | TGTAATACACACAGATCAAGGCTCACCACAGGGT | R-sc_0021 | ACGGTTCAAACAGTGAGAGATGGAAGGGGTATTCA | 1358 | N |
| scaffold_0027 | 1,538 | 25,040 | 2,245 | F-sc_0027 | TTGCTGTGGACACTCCAGCACGCCCAAATA | R-sc_0027 | AGAGGCACCAGCCGGAACAGCAACTGTAAC | 488 | N |
| Scaffold_0031 | 1,444 | 26,554 | 2,537 | F-sc_0031 | AACACCGTTAGCTCATGGAATTGCTCCAAAAACGTA | R-sc_0031 | TGCCGGACTTAAAGCCGCATTTGCCTCAGA | 589 | N |
| Scaffold_0034 | 1,382 | 47,949 | 4,824 | F-sc_0034 | GCCCTCAGCGCCAAATAGCAAGCCAAAACC | R-sc_0034 | ATGTGGCCTTGGGTGTTGGTGCTGGTGTAA | 747 | N |
| scaffold_0036 | 1,241 | 13,963 | 1,566 | F-sc_0036 | GTCCCAACGGTTGATGACCAACGCCACTCT | R-sc_0036 | GGGCGGACTTCGGTGCTAACCCAGAACAAG | 823 | N |
| Scaffold_0041 | 1,106 | 18,684 | 2,307 | F-sc_0041 | AGGAGGTGACAGACTAGGGTCTGGAAACAAACA | R-sc_0041 | TCCTGGTAAGCCTACCTTTGTCATGAATGGAACCAA | 148 | N |
| Scaffold_0050 | 950 | 46,126 | 6,583 | F-sc_0050 | AGCGGGAATGTTAGAAGCTGGGGACGGATT | R-sc_0050 | TTCCCACCCTCCGGGTGATATTCTCTCTCC | 164 | d |
| scaffold_0053 | 912 | 11,236 | 1,692 | F-sc_0053 | ACTGGAGCAGAAGGCTTAAGTTTAAAACATGGCCGA | R-sc_0053 | TGCGGCACTTCGTAAAACTTCGCGCATTGA | 300 | D |
| Scaffold_0054 | 911 | 82,250 | 12,465 | F-sc_0054 | GGAGCGGTGAAGGAGTTTGTGATTTGGATGGT | R-sc_0054 | CCGGGTTAAGGAGTTGTAGGCGCGGAGAAT | 342 | N |
| Scaffold_0059 | 856 | 35,258 | 5,317 | F-sc_0059 | CACCTCGCGGTGCGTCTTTTAGTTGGTGTA | R-sc_0059 | TGCTATCAACATTACCGTCATTTCCCTCACCTCCT | 103 | N |
| scaffold_0065 | 813 | 38,802 | 6,577 | F-sc_0065 | TGTCACTTGCAGGAGGCACAATGAGGGGAA | R-sc_0065 | ACACCACCACCGCGTGATAACCTACCATGT | 553 | D |
| Scaffold_0066 | 806 | 25,947 | 4,388 | F-sc_0066 | ACCAACAAGTGTTTGAACGACTAAGCAAGGCGA | R-sc_0066 | ACGAAGTGCAAAAGGCGGGCAAAGTCTAGT | 185 | D |
| Scaffold_0068 | 800 | 27,063 | 4,579 | F-sc_0068 | GAACAGCTGCCTTGCGCCGCTTCTCTCTTA | R-sc_0068 | CCCACCGCTTTTCGGCAAACATGATGCGAC | 460 | D |
| Scaffold_0076 | 770 | 35,673 | 6,469 | F-sc_0076 | GCGACGTAGGAGCGATTGCCTATTGAGGCT | R-sc_0076 | TCCTTTTACACTCTCACACCACGTGCGGGC | 123 | N |
| Scaffold_0077 | 768 | 87,025 | 15,462 | F-sc_0077 | TGATGATGCCAATGACGTTGTGGTGATGCCA | R-sc_0077 | AGCAGGATCTCCACCAGTAATAATGGAATTTGGGGA | 195 | N |
| Scaffold_0080 | 750 | 67,682 | 12,486 | F-sc_0080 | TGTGTCTTCTAGGTCTTGCGCTGCGGAGTC | R-sc_0080 | ACCAACAAACAAATACGGCACATGCGCACAA | 250 | N |
| scaffold_0088 | 723 | 21,957 | 4,212 | F-sc_0088 | GGTGCCAACTGGCATGAAGGCTGGTGTTTT | R-sc_0088 | AAACCAACACCCCCAGCAGCAGACTTTGGA | 167 | N |
| Scaffold_0090 | 710 | 103,387 | 20,038 | F-sc_0090 | TCCTAGGGTTCTCGCTTCCGCTCGGTCAAA | R-sc_0090 | CAAGGGAGAGGGTTCTAGCCCCACGACAGA | 404 | N |
| Scaffold_0091 | 701 | 22,077 | 4,369 | F-sc_0091 | ACGGGTAAGTGTCAAGAGACCCAAAACCCAGA | R-sc_0091 | ACATACGCTGTTGCGTGTGTGGTGGGACAA | 515 | N |
| ***Manually designed primers*** | | | | | | | | | |
| scaffold_0029 | 1,496 | 25,766 | 2,221 | F-sc_0029 | CTGAAGGTAAGTATTCAACTGAACGTCACATAG | R-sc_0029 | CCAAAGTTCTTCCAAACCTAGTGGATATGTGTCAGG | 347 | B |
| scaffold_0038 | 1,217 | 17,112 | 1,911 | F-sc_0038 | GATATGATAATTTGGCACAAGTAATCTACCTGAAGTTACC | R-sc_0038 | CAAAGATGTGTTGTCAAATATTTCACATTATACTCCGCTACC | 755 | D |
| scaffold_0044 | 938 | 11,146 | 1,654 | F-sc_0044 | CGGATTAATTGAAAGTCTATATACGGATTAATTGAAAGGAAAGCG | R-sc_0044 | CAATTAATCCGTCTTCAGCTTCTAATATTCCCGC | 552 | B |
| scaffold_0046 | 1,025 | 15,142 | 2,037 | F-sc_0046 | GATATGCTCCTTCTATTGACAGAATAGATGATTATAAACC | R-sc_0046 | ACTCATCTCTCCCATAATACTTTATAACTTTTGCTTGTCC | 373 | N |
| scaffold_0047 | 993 | 32,733 | 4,551 | F-sc_0047 | GCTGTTGCAGGTGTATACATAAGTCTTAATACCC | R-sc_0047 | AGTGTGAGTGGGCGAACACATGCAGGTG | 333 | B |
| scaffold_0048 | 972 | 53,853 | 6,909 | F-sc_0048 | CGCCACAGGTAGCCAGTGCATATCC | R-sc_0048 | CATCAAGCCAATCAGGATATGCGTTAGCTACC | 318 | N |
| scaffold_0062 | 832 | 11,790 | 1,952 | F-sc_0062 | CATATATCACACAAACAAACCAACACAGAAAGCC | R-sc_0062 | TCTGGCAAGACCTCTGCCCGAGTA | 224 | N |
| scaffold_0069 | 797 | 104,706 | 17,621 | F-sc_0069 | GATTATAAGATTTCATCTCAACTTCTTTAAGG | R-sc_0069 | GCTAAAAAAGTATATGAAGAAACTATTAATCATGG | 317 | N |
| scaffold_0081 | 746 | 37,439 | 6,724 | F-sc_0081 | CCTAATTCTGCTTCAAACTCTTCTAAAGAAATAGG | R-sc_0081 | TCTAATGAATTAGCTAAATTAGAAGGTGGTGTAACTCC | 382 | N |
| scaffold_0089 | 715 | 9,986 | 1,934 | F-sc_0089 | CAATTCTATATGTTTATATACAATTGTATGTTG | R-sc_0089 | AGGAAGATTAGAGATGGGTCGTGTAGCGTAGCGG | 331 | N |
| scaffold_0099 | 643 | 81,487 | 16,917 | F-sc_0099 | CTACATTATCAGAAGCTTCACTAGTCATTACATC | R-sc_0099 | CTAGCTATCAAAAATGCTAAAATAATAAAGAATATGG | 275 | N |
| scaffold_0100 | 640 | 5,705 | 1,255 | F-sc_0100 | GAGATGATATTGAAGACCAACGAAACG | R-sc_0100 | CCGGAGGTTGAGGGTAATACTATCC | 258 | N |
| scaffold_0104 | 492 | 9,504 | 2,619 | F-sc_0104 | CATGAGTTTGTATGTGCGTGAGTGTGTATGTTCG | R-sc_0104 | TGTATGCGCGTGAGTGTGCATGTGCG | 398 | B |
| scaffold_0107 | 612 | 95,237 | 21,033 | F-sc_0107 | AGCTTTCTTAAAACTAGCTCTACGGACGTAGT | R-sc_0107 | GGTAAGAACATCCAAAAAGTTAGGGCTATC | 273 | N |
| scaffold_0117 | 574 | 2,913 | 675 | F-sc_0117 | GCAGAGAATGAATATAGAGTAGAAATGG | R-sc_0117 | GAACCCTAGGAAGTTACCGAATGG | 272 | N |
| scaffold_0120 | 566 | 26,316 | 6,091 | F-sc_0120 | GTGCACGAGAGTATGAAAGATACAG | R-sc_0120 | CTTTTCTTTGGTTTTTGACCGAGCGG | 203 | B |
| scaffold_0124 | 425 | 24,817 | 8,205 | F-sc_0124 | ACTTCCCAGAATGGAATAACGGCACC | R-sc_0124 | TCTTTGTCTGCGGGGATGCTTACTGCTTTGG | 351 | B |
| scaffold_0165 | 442 | 6,727 | 1,727 | F-sc_0165 | GTTGTTTCCTGGATGTTAACAGTTGTTTCACTATTG | R-sc_0165 | GTCAACTATGGATGAAATGTACATTTGTTGATGG | 298 | N |
| ***Primers for RT-PCR*** | | | | | | | | | |
| contig_01 | 507 | 1,169,291 | 272,143 | F-RT-contig_01 | AGTCTGGTGCCAGCAGCCGCGGTAA | R-RT-contig_01 | TGGCAAGGGGGACGACACCGCGCA | 151 | B |
| contig_02 | 1,078 | 1,716,902 | 187,935 | F-RT-contig_02 | TGGCAGTGGGGCTTCCCGCGTGCGT | R-RT-contig_02 | TGCGCCCCGCCCGAGGACGA | 269 | B |
| contig_03 | 591 | 2,663,373 | 531,773 | F-RT-contig_03 | TGGATGGCGCTAGAGCGTCGGGCCT | R-RT-contig_03 | TGCACCCGAGGCGGCTCCACTCAGGCT | 147 | B |
| contig_06 | 937 | 1,318,363 | 166,027 | F-RT-contig_06 | CGCGCGGCGCCATTTCGGTGGCA | R-RT-contig_06 | AGCGCGCGTGCGGCCCTGGA | 116 | N |

Mean coverage=Number of reads×Average read length/Scaffold length

D: Amplification specific to diseased abalone, N: No amplification, B: Amplification in both abalone species, d: Amplification in only one diseased abalone species

F: forward, R: reverse

Supplemental Table 2. Primers used for gap closing

| Scaffold | F-primer | R-primer |
|---|---|---|
| Scaffold-1 | GTGGTCTTGATTACATGACACAGTTAAAATTAGGGCATG | CGGGTCAATCCGTGGTATCTTTGATGCGTGTTCTAC |
| Scaffold-4 | GCGGTGTTTTAGTTATTTCATTAAGTCCAATTTGGTGATACC | CTCGAAAATGTCTTTATGTCGATTCCACGAAATTACACATTACC |
| Scaffold-5 | CAGTGAAAAATTCCATCAACCGATCACTTGTTGACC | CCATACGAACAAACCCATTTAGTGAACGCCGCCATACAATCTGG |
| Scaffold-7 | CCGTCGAAATTACAAAACCTATCACGTCTCGGC | GTTTTTCTTAACCATCACCACATTAATCACCTGCTCTGAAGGC |
| Scaffold-8 | CCTAGACCGATTAGTGATACATTCCATTCACGGC | GTCCGACGGAATATCCTTCTGGAAGTCGGAG |
| Scaffold-9 | CAATTATGGATTACAAAACATGCAGTGGCGCGGACACC | GTCGGAGGATCAAGTGTTTGAACCAAAGATGGCTC |
| Scaffold-11 | GTGGATTAATATTTGCGCCAATCCCTAATTTACCAAGTGCCG | CATAGCAAAAGGACAGTTATAAAAATCGGGAAATTGTGCGG |
| Scaffold-15 | CTTCAAACACATTTATATCACGTGTTGCGGTGATTTGGCC | CCAAAAGATACAGTCTCATTATATATTGCTTATCCCG |
| Scaffold-38 | GGTTCCACATGGTAAACATTTTCTAGCAACACTTGATGG | GGATACGGTAACTTCAGGTAGATTACTTGTGCC |
| Scaffold-53 | CACCGTGATATCGCTATTGATATGACTGCAGCAG | CCAGAAATAATAGCATAAGATTGATGATCGTGTGAGTC |
| Scaffold-65 | GACATGGGGCAAAAAAGAATTATAAATATAGCGGAGCC | CTCTTTCGCGCTCATATCAATTTCACCAGTCATTTGCC |
| Scaffold-66 | CATGTAATTTATATCAAAAAAACTAGACTTTGCCCGCC | CCTATACCTGGTGAAACATTTGCAGAACTGGTTG |
| Scaffold-68 | TTTGCCGAAAAGCGGTGGGCAAATAACTGG | GGAAAAGTAAAATATAAATTCGATCGCCAACTTAGTATTGG |

F: forward, R: reverse

Supplemental Table 3. Characterization of potential open reading frames of AbAV genome

| | | | | | Best BLASTP hits of AbAV ORF against virus database | | | | | | | |
|---|---|---|---|---|---|---|---|---|---|---|---|---|
| ORF [a] | Nucleotide position | | Orientation [b] | Length (codon | Predicted similarity and/or function | Species | Family | Accession no. [c] | Total score | Query cover | E-value | Identity |
| ORF-1 | 2 | 970 | + | 322 | | | | | | | | |
| ORF-2 | 1005 | 2846 | + | 613 | | | | | | | | |
| ORF-3 | 2864 | 4534 | + | 556 | | | | | | | | |
| ORF-4 | 4562 | 6229 | + | 555 | | | | | | | | |
| ORF-5 | 6221 | 6709 | - | 162 | YbiA | Stenotrophomonas phage DLP4 | Siphoviridae | ATS92227.1 | 122 | 88% | 4.00E-34 | 42.36% |
| ORF-6 | 6772 | 7254 | + | 160 | | | | | | | | |
| ORF-7 | 7297 | 7791 | + | 164 | | | | | | | | |
| ORF-8 | 7842 | 8258 | + | 138 | | | | | | | | |
| ORF-9 | 8308 | 8745 | + | 145 | | | | | | | | |
| ORF-10 | 9256 | 9588 | + | 110 | | | | | | | | |
| ORF-11 | 9663 | 10109 | + | 148 | | | | | | | | |
| ORF-12 | 10162 | 10566 | + | 134 | | | | | | | | |
| ORF-13 | 10601 | 11005 | + | 134 | | | | | | | | |
| ORF-14 | 11042 | 11524 | + | 160 | | | | | | | | |
| ORF-15 | 11576 | 12088 | + | 170 | | | | | | | | |
| ORF-16 | 12085 | 13371 | - | 428 | inhibition of apoptosis protein (IAP-3) | Spodoptera frugiperda multiple nucleopolyhedrovirus | Baculoviridae | ACA02668.1 | 55.8 | 20% | 9.00E-07 | 34.09% |
| ORF-17 | 13438 | 13731 | + | 97 | Viral histone-like protein; A104R | African swine fever virus | Asfarviridae (NCLDV) | P0C9E4.1 | 75.1 | 98% | 3.00E-17 | 41.67% |
| ORF-18 | 13755 | 14435 | + | 226 | | | | | | | | |
| ORF-19 | 14415 | 15434 | - | 339 | Uncharacterized protein F317L | African swine fever virus | Asfarviridae (NCLDV) | P0CAE0.1 | 87 | 71% | 2.00E-17 | 24.00% |
| ORF-20 | 15572 | 16564 | - | 330 | Ribonucleotide reductase small subunit | African swine fever virus | Asfarviridae (NCLDV) | AIY22235.1 | 269 | 98% | 4.00E-87 | 45.90% |
| ORF-21 | 16595 | 18979 | + | 794 | F778R | African swine fever virus | Asfarviridae (NCLDV) | AXZ95821.1 | 559 | 93% | 0 | 41.10% |
| ORF-22 | 18971 | 22060 | - | 1029 | Putative helicase/primase complex protein;pF1055L | African swine fever virus | Asfarviridae (NCLDV) | P0CA11.1 | 525 | 97% | 1.00E-167 | 34.70% |
| ORF-23 | 22117 | 22389 | + | 90 | | | | | | | | |
| ORF-24 | 22416 | 22589 | + | 57 | | | | | | | | |
| ORF-25 | 22622 | 22921 | + | 99 | | | | | | | | |
| ORF-26 | 22928 | 23071 | + | 47 | | | | | | | | |
| ORF-27 | 23074 | 23229 | + | 51 | | | | | | | | |
| ORF-28 | 23268 | 23540 | + | 90 | | | | | | | | |
| ORF-29 | 23601 | 23729 | + | 42 | | | | | | | | |
| ORF-30 | 23937 | 24119 | + | 60 | | | | | | | | |
| ORF-31 | 24148 | 24351 | + | 67 | | | | | | | | |
| ORF-32 | 24373 | 24516 | + | 47 | | | | | | | | |
| ORF-33 | 24545 | 24709 | + | 54 | | | | | | | | |
| ORF-34 | 24742 | 25788 | + | 348 | | | | | | | | |
| ORF-35 | 25820 | 26530 | - | 236 | | | | | | | | |
| ORF-36 | 26572 | 27795 | + | 407 | Probable methyltransferase, EP424R | African swine fever virus | Asfarviridae (NCLDV) | AJL34069.1 | 213 | 89% | 6.00E-63 | 38.36% |
| ORF-37 | 27834 | 28658 | + | 274 | | | | | | | | |
| ORF-38 | 28705 | 28971 | + | 88 | | | | | | | | |
| ORF-39 | 29004 | 29579 | + | 191 | thymidine kinase | NY_014 poxvirus | poxvirus (NCLDV) | YP_009408474.1 | 123 | 96% | 9.00E-34 | 34.24% |
| ORF-40 | 29949 | 31238 | + | 429 | K421R | African swine fever virus | Asfarviridae (NCLDV) | AXZ95828.1 | 186 | 96% | 2.00E-52 | 30.57% |
| ORF-41 | 31189 | 34902 | - | 1237 | RNA polymerase subunit 2 | African swine fever virus | Asfarviridae (NCLDV) | CAN10153.1 | 1673 | 98% | 0 | 64.98% |
| ORF-42 | 34990 | 35913 | + | 307 | | | | | | | | |
| ORF-43 | 35935 | 36381 | + | 148 | | | | | | | | |
| ORF-44 | 36404 | 37339 | + | 311 | pEP364R | African swine fever virus | Asfarviridae (NCLDV) | AXB49288.1 | 97.8 | 96% | 3.00E-21 | 27.08% |
| ORF-45 | 37336 | 41244 | - | 1302 | BA71V-M1249L | African swine fever virus | Asfarviridae (NCLDV) | P0CAB5.1 | 442 | 78% | 2.00E-132 | 29.82% |
| ORF-46 | 41286 | 42533 | + | 415 | pM448R | African swine fever virus | Asfarviridae (NCLDV) | AXB49290.1 | 102 | 71% | 1.00E-21 | 26.54% |
| ORF-47 | 42552 | 42851 | + | 99 | | | | | | | | |
| ORF-48 | 42848 | 43192 | - | 114 | Uncharacterized protein C129R | African swine fever virus | Asfarviridae (NCLDV) | P0CA38.1 | 93.6 | 94% | 5.00E-24 | 41.96% |
| ORF-49 | 43218 | 43403 | - | 61 | | | | | | | | |
| ORF-50 | 43450 | 45501 | + | 683 | Uncharacterized protein C717R | African swine fever virus | Asfarviridae (NCLDV) | P0CAJ6.1 | 307 | 96% | 1.00E-92 | 31.61% |
| ORF-51 | 45524 | 45841 | + | 105 | Uncharacterized protein C122R | African swine fever virus | Asfarviridae (NCLDV) | P0CA22.1 | 94 | 95% | 1.00E-24 | 44.00% |
| ORF-52 | 45843 | 46349 | - | 168 | | | | | | | | |
| ORF-53 | 46401 | 47789 | - | 462 | Putative poly(A) polymerase catalytic subunit | African swine fever virus | Asfarviridae (NCLDV) | P0C9D2.1 | 258 | 100% | 6.00E-79 | 34.16% |
| ORF-54 | 47822 | 48841 | + | 339 | Uncharacterized protein C315R | African swine fever virus | Asfarviridae (NCLDV) | P0CAD6.1 | 149 | 93% | 2.00E-40 | 31.88% |
| ORF-55 | 48836 | 49255 | - | 139 | | | | | | | | |
| ORF-56 | 49369 | 49596 | - | 75 | | | | | | | | |
| ORF-57 | 49571 | 51829 | - | 752 | BA71V-B962L | African swine fever virus | Asfarviridae (NCLDV) | AJL34086.1 | 624 | 98% | 0 | 45.21% |
| ORF-58 | 51838 | 52203 | - | 121 | FAD-linked sulfhydryl oxidase (p14) | African swine fever virus | Asfarviridae (NCLDV) | Q9JFM9.1 | 86.7 | 79% | 3.00E-21 | 43.75% |
| ORF-59 | 52208 | 53632 | - | 474 | | | | | | | | |
| ORF-60 | 53673 | 54137 | - | 154 | | | | | | | | |
| ORF-61 | 54145 | 54903 | - | 252 | hypothetical protein D5b_00348 | Faustovirus | Asfaviridae ? (NCLDV) | AMN83769.1 | 111 | 88% | 1.00E-26 | 31.14% |
| ORF-62 | 54905 | 56056 | - | 383 | B354L | African swine fever virus | Asfarviridae (NCLDV) | AXZ95853.1 | 271 | 98% | 1.00E-86 | 40.00% |
| ORF-63 | 56071 | 56556 | - | 161 | | | | | | | | |
| ORF-64 | 56587 | 57114 | - | 175 | | | | | | | | |
| ORF-65 | 57107 | 57646 | - | 179 | | | | | | | | |
| ORF-66 | 57715 | 59334 | - | 539 | BA71V-B602L (9RL) | African swine fever virus | Asfarviridae (NCLDV) | AJL34093.1 | 240 | 97% | 9.00E-71 | 29.53% |
| ORF-67 | 59371 | 60480 | + | 369 | Zinc finger protein B385R | African swine fever virus | Asfarviridae (NCLDV) | Q8V9S7.1 | 244 | 93% | 6.00E-76 | 39.89% |
| ORF-68 | 60482 | 62425 | - | 647 | major capsid protein p72 | African swine fever virus | Asfarviridae (NCLDV) | AAT84439.1 | 583 | 99% | 0 | 44.99% |
| ORF-69 | 62439 | 62888 | - | 149 | | | | | | | | |
| ORF-70 | 62906 | 64078 | - | 390 | Hypothetical protein PACV_238 | Pacmanvirus A23 | Asfaviridae ? (NCLDV) | YP_009361587.1 | 137 | 91% | 4.00E-35 | 28.49% |
| ORF-71 | 64121 | 64741 | - | 206 | BA71V-B175L | African swine fever virus | Asfarviridae (NCLDV) | AJL34264.1 | 97.1 | 83% | 1.00E-23 | 31.40% |
| ORF-72 | 64824 | 65612 | + | 262 | B263R | African swine fever virus | Asfarviridae (NCLDV) | AXZ95861.1 | 171 | 98% | 1.00E-50 | 40.91% |
| ORF-73 | 65597 | 65941 | - | 114 | | | | | | | | |
| ORF-74 | 65964 | 70244 | - | 1426 | BA71V-B175L | African swine fever virus | Asfarviridae (NCLDV) | AJL34102.1 | 770 | 99% | 0 | 34.52% |
| ORF-75 | 70289 | 70861 | + | 190 | hypothetical protein D6_00329 | Faustovirus | Asfaviridae ? (NCLDV) | AMN84731.1 | 120 | 91% | 2.00E-32 | 37.56% |
| ORF-76 | 70882 | 74226 | + | 1114 | DNA polymerase beta | African swine fever virus | Asfarviridae (NCLDV) | P0C972.1 | 748 | 96% | 0 | 39.60% |
| ORF-77 | 74257 | 74790 | + | 177 | | | | | | | | |
| ORF-78 | 74785 | 75675 | - | 296 | putative ubiquitin carboxyl-terminal hydrolase 2-like | Harvfovirus sp. | Mimiviridae (NCLDV) | AYV80395.1 | 55.1 | 86% | 8.00E-07 | 23.88% |
| ORF-79 | 75656 | 75904 | - | 82 | | | | | | | | |
| ORF-80 | 75907 | 83604 | - | 2565 | CP2475L (p220) | African swine fever virus | Asfarviridae (NCLDV) | AXZ95866.1 | 981 | 99% | 0 | 29.29% |
| ORF-81 | 83681 | 85270 | + | 529 | 60 kDa polyprotein | African swine fever virus | Asfarviridae (NCLDV) | AIY22285.1 | 325 | 95% | 1.00E-103 | 39.85% |
| ORF-82 | 85284 | 85535 | + | 83 | Putative DNA-directed RNA polymerase subunit 10 homolog | African swine fever virus | Asfarviridae (NCLDV) | P0C979.1 | 69.7 | 100% | 1.00E-15 | 42.86% |
| ORF-83 | 85547 | 86647 | + | 366 | CP312R | African swine fever virus | Asfarviridae (NCLDV) | AYW34067.1 | 135 | 83% | 4.00E-35 | 31.68% |
| ORF-84 | 86667 | 91013 | - | 1448 | Probable DNA-directed RNA polymerase subunit 1 homolog | African swine fever virus | Asfarviridae (NCLDV) | P0C988.1 | 1637 | 99% | 0 | 53.96% |
| ORF-85 | 91071 | 92453 | - | 460 | Chain A, DNA ligase | African swine fever virus | Asfarviridae (NCLDV) | AXZ95874.1 | 256 | 92% | 4.00E-79 | 37.76% |
| ORF-86 | 92512 | 93390 | - | 292 | | | | | | | | |
| ORF-87 | 93442 | 94488 | - | 348 | | | | | | | | |
| ORF-88 | 94588 | 95010 | - | 140 | | | | | | | | |
| ORF-89 | 95041 | 95601 | - | 186 | | | | | | | | |
| ORF-90 | 95651 | 96196 | - | 181 | | | | | | | | |
| ORF-91 | 96247 | 96498 | - | 83 | | | | | | | | |
| ORF-92 | 96528 | 96686 | - | 52 | | | | | | | | |
| ORF-93 | 96727 | 97017 | - | 96 | | | | | | | | |
| ORF-94 | 97055 | 97543 | - | 162 | | | | | | | | |
| ORF-95 | 97575 | 98027 | - | 150 | | | | | | | | |
| ORF-96 | 98082 | 98441 | + | 119 | | | | | | | | |
| ORF-97 | 98470 | 98766 | + | 98 | | | | | | | | |
| ORF-98 | 98761 | 99768 | - | 335 | | | | | | | | |
| ORF-99 | 99831 | 100247 | + | 138 | | | | | | | | |
| ORF-100 | 100251 | 100697 | - | 148 | | | | | | | | |
| ORF-101 | 100798 | 102825 | + | 675 | | | | | | | | |
| ORF-102 | 103488 | 103751 | - | 87 | | | | | | | | |
| ORF-103 | 103786 | 104781 | + | 331 | | | | | | | | |
| ORF-104 | 104796 | 105665 | + | 289 | Transcription factor S-II-related protein | African swine fever virus | Asfarviridae (NCLDV) | P0C8F8.1 | 123 | 67% | 7.00E-32 | 39.30% |
| ORF-105 | 105662 | 105841 | - | 59 | | | | | | | | |
| ORF-106 | 105893 | 106333 | + | 146 | | | | | | | | |
| ORF-107 | 106395 | 106610 | + | 71 | | | | | | | | |
| ORF-108 | 106638 | 108146 | + | 502 | hypothetical protein BpV2_110c | Bathycoccus sp. RCC1105 virus BpV2 | Phycodnaviridae (NCLDV) | ADQ91277.1 | 137 | 92% | 1.00E-33 | 24.03% |
| ORF-109 | 108259 | 109539 | + | 426 | hypothetical protein Hokovirus_3_43 | Hokovirus HKV1 | Mimiviridae (NCLDV) | ARF10770.1 | 94.7 | 53% | 1.00E-04 | 24.66% |
| ORF-110 | 109669 | 110100 | + | 143 | | | | | | | | |
| ORF-111 | 110254 | 110820 | - | 188 | | | | | | | | |
| ORF-112 | 110861 | 112117 | + | 418 | | | | | | | | |
| ORF-113 | 112168 | 112581 | + | 137 | | | | | | | | |
| ORF-114 | 112586 | 113095 | - | 169 | | | | | | | | |
| ORF-115 | 113151 | 113747 | - | 198 | | | | | | | | |
| ORF-116 | 113790 | 114314 | - | 174 | dUTP diphosphatase | Pithovirus LCPAC101 | Pithoviridae (NCLDV) | QBK90048.1 | 73.6 | 84% | 6.00E-15 | 35.48% |
| ORF-117 | 114370 | 114939 | + | 189 | E199L | African swine fever virus | Asfarviridae (NCLDV) | AZP54233.1 | 72.4 | 59% | 5.00E-14 | 34.75% |
| ORF-118 | 114970 | 115362 | + | 130 | Uncharacterized protein E146L | African swine fever virus | Asfarviridae (NCLDV) | P0CA54.1 | 48.5 | 83% | 3.00E-06 | 30.77% |
| ORF-119 | 115349 | 116317 | - | 322 | Uncharacterized protein E301R | African swine fever virus | Asfarviridae (NCLDV) | Q65239.2 | 236 | 87% | 1.00E-74 | 45.26% |
| ORF-120 | 116336 | 117679 | - | 447 | | | | | | | | |
| ORF-121 | 117708 | 118328 | + | 206 | | | | | | | | |
| ORF-122 | 118366 | 118806 | + | 146 | | | | | | | | |
| ORF-123 | 118785 | 119348 | + | 187 | | | | | | | | |
| ORF-124 | 119305 | 120405 | - | 366 | Cysteine desulfurase NifS | Pacmanvirus A23 | Asfaviridae ? (NCLDV) | YP_009361727.1 | 125 | 73% | 1.00E-30 | 31.83% |
| ORF-125 | 120409 | 121917 | - | 502 | Chain A, Major Capsid Protein | Faustovirus | Asfaviridae ? (NCLDV) | 5J7O_A | 201 | 99% | 1.00E-55 | 25.19% |
| ORF-126 | 121953 | 124970 | + | 1005 | helicase Q706 | African swine fever virus | Asfarviridae (NCLDV) | NP_042814.1 | 596 | 67% | 5.00E-110 | 46.53% |
| ORF-127 | 124951 | 125757 | - | 268 | pH240R | African swine fever virus | Asfarviridae (NCLDV) | NP_042812.1 | 69.3 | 95% | 5.00E-12 | 26.64% |
| ORF-128 | 125779 | 126252 | - | 157 | Hypothetical protein PACV_339 | Pacmanvirus A23 | | YP_009361686.1 | 42.7 | 28% | 0.001 | 42.22% |
| ORF-129 | 126279 | 127400 | - | 373 | pH339R | African swine fever virus | Asfarviridae (NCLDV) | Q65232.1 | 181 | 89% | 3.00E-52 | 33.63% |
| ORF-130 | 127436 | 127864 | - | 142 | | | | | | | | |
| ORF-131 | 127906 | 128199 | - | 97 | | | | | | | | |
| ORF-132 | 128242 | 129423 | + | 393 | RNA polymerase subunit 3 | African swine fever virus | Asfarviridae (NCLDV) | NP_042806.1 | 162 | 98% | 2.00E-44 | 29.67% |
| ORF-133 | 129413 | 132922 | - | 1169 | DNA topoisomerase 2 | African swine fever virus | Asfarviridae (NCLDV) | AJL34125.1 | 976 | 99% | 0 | 43.36% |
| ORF-134 | 132955 | 133062 | - | 35 | | | | | | | | |
| ORF-135 | 133071 | 133961 | - | 296 | Cysteine protease S273R | African swine fever virus | Asfarviridae (NCLDV) | P0C9B7.1 | 244 | 95% | 2.00E-78 | 43.21% |
| ORF-136 | 133974 | 134597 | + | 207 | | | | | | | | |
| ORF-137 | 134606 | 135619 | + | 337 | Uncharacterized protein D345L | African swine fever virus | Asfarviridae (NCLDV) | Q65225.2 | 127 | 90% | 3.00E-32 | 27.94% |
| ORF-138 | 136332 | 136670 | + | 112 | | | | | | | | |
| ORF-139 | 136713 | 136865 | + | 50 | | | | | | | | |
| ORF-140 | 136905 | 137075 | + | 56 | | | | | | | | |
| ORF-141 | 137084 | 140569 | + | 1161 | BA71V-D1133L (g10L) | African swine fever virus | Asfarviridae (NCLDV) | AJL34119.1 | 1002 | 99% | 0 | 45.91% |
| ORF-142 | 140603 | 141544 | + | 313 | BA71V-D339L (g9L) | African swine fever virus | Asfarviridae (NCLDV) | AJL34283.1 | 91.3 | 65% | 5.00E-19 | 30.48% |
| ORF-143 | 141570 | 141812 | + | 80 | | | | | | | | |
| ORF-144 | 141841 | 142434 | + | 197 | | | | | | | | |
| ORF-145 | 142446 | 142955 | + | 169 | | | | | | | | |
| ORF-146 | 142935 | 143876 | - | 313 | mRNA-decapping protein g5R | African swine fever virus | Asfarviridae (NCLDV) | Q65217.1 | 84.7 | 76% | 3.00E-17 | 25.82% |
| ORF-147 | 143957 | 144472 | + | 171 | | | | | | | | |
| ORF-148 | 144639 | 145340 | + | 233 | | | | | | | | |
| ORF-149 | 145374 | 146102 | + | 242 | | | | | | | | |
| ORF-150 | 146130 | 146870 | + | 246 | | | | | | | | |
| ORF-151 | 146891 | 147316 | + | 141 | | | | | | | | |
| ORF-152 | 147355 | 148242 | + | 295 | | | | | | | | |
| ORF-153 | 148274 | 148828 | + | 184 | | | | | | | | |
| ORF-154 | 148858 | 149313 | + | 151 | | | | | | | | |
| ORF-155 | 149356 | 149835 | + | 159 | | | | | | | | |
| ORF-156 | 149868 | 150344 | + | 158 | | | | | | | | |
| ORF-157 | 150337 | 152808 | - | 823 | pNP868R guanylyltransferase | African swine fever virus | Asfarviridae (NCLDV) | AIY22450.1 | 464 | 96% | 3.00E-149 | 34.63% |
| ORF-158 | 152849 | 154783 | - | 644 | tail fiber protein | Synechococcus phage S-B28 | Myoviridae | QBP05809.1 | 295 | 48% | 5.00E-14 | 38.57% |
| ORF-159 | 154819 | 155181 | - | 120 | | | | | | | | |

BLASTP homology search of AbAV ORFs against the viral database (*E-value*<1e-3).

ORFs were predicted using the RAST program.

a: Open reading frames in the AbAV genome were numbered sequentially.

b: The direction of the transcripts are indicated by + (right) or - (left).

c: GenBank accession numbers of homologous proteins.
